# Myeloid Cells Expressing VEGF and Arginase-1 Following Uptake of Damaged Retinal Pigment Epithelium Suggests Potential Mechanism That Drives the Onset of Choroidal Angiogenesis in Mice

**DOI:** 10.1371/journal.pone.0072935

**Published:** 2013-08-16

**Authors:** Jian Liu, David A. Copland, Shintaro Horie, Wei-Kang Wu, Mei Chen, Yunhe Xu, B. Paul Morgan, Matthias Mack, Heping Xu, Lindsay B. Nicholson, Andrew D. Dick

**Affiliations:** 1 Unit of Ophthalmology, School of Clinical Sciences, University of Bristol, Bristol, United Kingdom; 2 Centre for Vision and Vascular Science, School of Medicine, Dentistry and Biomedical Sciences, Queen’s University Belfast, Belfast, United Kingdom; 3 School of Medicine, Cardiff University, Cardiff, United Kingdom; 4 Department of Internal Medicine II, University Hospital, Regensburg, Regensburg, Germany; 5 School of Cellular and Molecular Medicine, University of Bristol, Bristol, United Kingdom; Institut de la Vision, France

## Abstract

Whilst data recognise both myeloid cell accumulation during choroidal neovascularisation (CNV) as well as complement activation, none of the data has presented a clear explanation for the angiogenic drive that promotes pathological angiogenesis. One possibility that is a pre-eminent drive is a specific and early conditioning and activation of the myeloid cell infiltrate. Using a laser-induced CNV murine model, we have identified that disruption of retinal pigment epithelium (RPE) and Bruch’s membrane resulted in an early recruitment of macrophages derived from monocytes and microglia, prior to angiogenesis and contemporaneous with lesional complement activation. Early recruited CD11b^+^ cells expressed a definitive gene signature of selective inflammatory mediators particularly a pronounced *Arg-1* expression. Accumulating macrophages from retina and peripheral blood were activated at the site of injury, displaying enhanced VEGF expression, and notably prior to exaggerated VEGF expression from RPE, or earliest stages of angiogenesis. All of these initial events, including distinct VEGF ^+^ Arg-1^+^ myeloid cells, subsided when CNV was established and at the time RPE-VEGF expression was maximal. Depletion of inflammatory CCR2-positive monocytes confirmed origin of infiltrating monocyte *Arg-1* expression, as following depletion *Arg-1* signal was lost and CNV suppressed. Furthermore, our *in vitro* data supported a myeloid cell uptake of damaged RPE or its derivatives as a mechanism generating VEGF ^+^ Arg-1^+^ phenotype *in vivo*. Our results reveal a potential early driver initiating angiogenesis via myeloid-derived VEGF drive following uptake of damaged RPE and deliver an explanation of why CNV develops during any of the stages of macular degeneration and can be explored further for therapeutic gain.

## Introduction

Choroidal neovascularisation (CNV), angiogenesis arising from the extension of choriocapillaris into the subretinal space and toward the outer retina, accounts for severe visual impairment in patients with advanced age-related macular degeneration (AMD) [1]. The precise mechanisms that initiate the dominant VEGF drive of CNV in AMD are largely unknown, particularly on the background of several clinico-pathological features including complement activation, retinal pigment epithelium (RPE) dysfunction, drusen formation at the RPE/Bruch’s membrane (BM) interface and photoreceptor degeneration [2,3]. During the life-span of RPE, environmental factors, including long-term chronic light-oxidative stress and the accumulation of lipofuscin (A2E) and other oxidative lipids, lead to progressive RPE dysfunction and death, often associated with drusen deposition. Accordingly, RPE stress and degeneration occur prior to the ultimate geographic atrophy as well as CNV. CNV however may occur during any of the clinical stages of drusen formation whilst the disease marches toward geographic atrophy [2–5].

Although complement remains a widely accepted pathogenic pathway in AMD, the contribution of both innate and potentially adaptive immunity is also prominent [6]. In particular, one notion is that para-inflammation acts as a potential regulator of chronic tissue damage as seen in degenerative conditions whilst further activation of immune responses will drive a pathogenic chronic inflammation and tissue damage [7–9]. In ageing eyes, accumulation of myeloid cells, especially macrophages, alongside complement components adjacent to and within drusen, is observed throughout phases of the disease and in particular in large number at time of CNV [1,2]. The intrinsic nature of macrophages enables them to respond readily to the tissue environment, as shown with plasticity of immune cells recovered from the ocular compartments [9,10]. In mouse, the paradigm of M1 and M2 macrophage phenotype has been studied with respect to angiogenesis [11]. Both M1 macrophages (nitric oxide synthase 2, NOS2^+^) which are functionally pro-inflammatory, and M2 macrophages (Arginase-1, Arg-1^+^) - a phenotype conferring responses related to wound healing, are capable of generating VEGF and promoting angiogenesis. However, pathological angiogenesis is observed most commonly in the context of M2 macrophages [12–14]. Notwithstanding these data, clinical evidence demonstrates both macrophage phenotypes contribute at different stages of AMD [15,16].

Nevertheless, previous studies determining the mechanisms through which macrophages contribute to CNV have generated conflicting results, potentially due in part to the complex and kinetic microenvironment that governs macrophage phenotype and function [4,17–20]. Studies using transgenic mice advance inflammatory M1 phenotype monocyte (CCR2^+^) infiltrate as the drive for experimental CNV [17], whilst others have illuminated resident retinal macrophages (microglia) to contribute also to CNV pathology [21]. Whilst mechanisms have been proposed, none, until recently has delivered a driver of angiogenesis [19]. The purpose of this study was to characterise the source and phenotype of macrophages recruited to the site of injury in the laser-induced CNV model, to then assess what conditions the myeloid cell phenotype we observe, and finally explore to what extent that contributes to pathological angiogenesis. Our data provide new insights into the early events of laser induced CNV, and demonstrate a pivotal role for early accumulation of infiltrating myeloid cells which are rapidly conditioned via uptake of damaged RPE or its derivatives toward Arg-1 ^+^ VEGF^+^ phenotype. Such events occur prior to the onset of angiogenesis, overt complement damage and further RPE loss or exaggerated VEGF production from RPE.

## Results

### Laser photocoagulation induces focal RPE/BM injury, cell death and DNA damage

**Figure 1:**
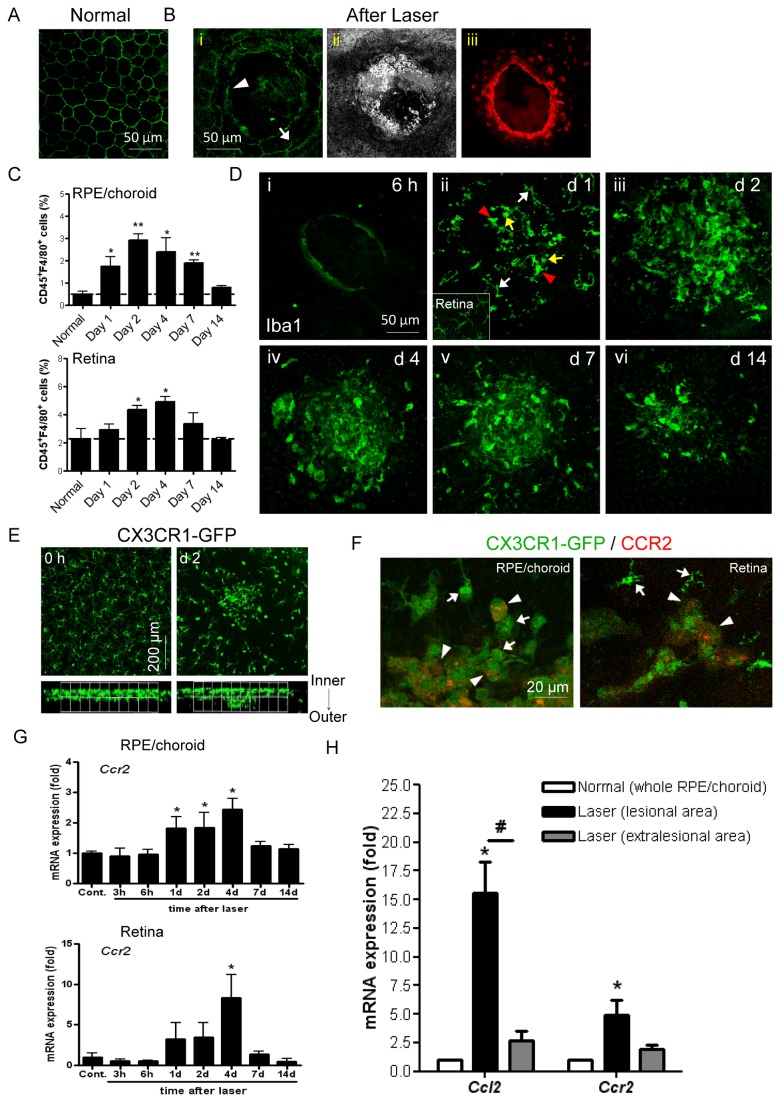
Early recruitment of retina- and blood-derived macrophages to site of RPE/BM injury. (**A**) Normal RPE shows typical pentagonal or hexagonal cell morphology examined on RPE/choroid whole-mounts using Claudin immunostaining and confocal microscopy. (**B**) Laser induction results in (**i**) disruption of RPE (arrow) and BM (arrowhead); (ii) tissue fragmentation; and (iii) TUNEL-positive cells with DNA damage contiguous as observed with confocal microscopic analysis of RPE on RPE/choroidal whole-mounts. (**C**) Flow cytometric analysis of pooled samples from three eyes shows CD45^+^F4/80^+^ cell infiltration in RPE/choroid and retina between 1–14 days post laser (mean ± SEM, n=2-4 for each time point). (**D**) Iba1 immunohistochemistry analysis shows local recruitment and morphological change of Iba1^+^ microglia/macrophages at site of injury at 6 hours (**i**), day 1 (ii), day 2 (iii), day 4 (iv), day 7 (v) and day 14 (vi) after laser treatment. Small image inset in (**D**, **ii**) shows ramified microglia in normal retina. (**E**) Top and side view of Z-stacks of confocal images of retina whole-mounts collected from CX3CR1^*gfp/+*^ mice immediately after laser induction or 2 days after. (**F**) CCR2 immunohistochemistry of RPE/choroid or retina from CX3CR1^*gfp/+*^ mice demonstrates accumulation of CX3CR1 ^hi^CCR2^-^ (microglia-derived, **arrow**) and CX3CR1 ^lo^CCR2^+^ (monocyte-derived, **arrowhead**) macrophages at lesion site on both RPE and outer retina aspects. (**G**) QRT-PCR analysis of laser-induced *Ccr2* mRNA expression in RPE/choroid and retina tissues over time. mRNA levels were normalised against a house keeping gene *Gapdh* and data are presented as fold change relative to control (mean ± SEM, n=3-6 for each time point). (**H**) Local *Ccl2* and *Ccr2* upregulation at the RPE/choroid lesions (day 2). ^*^ *P*<0.05 vs. control; ^**^ *P*<0.01 vs. control; ^#^ *P*<0.05 between groups.

Laser-induced CNV model is an accelerated model resembling in part pathogenic processes for neovascular AMD, by which laser photocoagulation ruptures RPE/BM in animals, mimics acute RPE dysfunction and cell loss found in human ageing eyes [22,23]. We wished to initially determine the extent of damage to RPE that may elicit microglia or myeloid cell responses to drive pathological angiogenesis. Immunostaining for the tight junction protein, Claudin, on whole-mounts of normal RPE/choroid showed typical pentagonal or hexagonal RPE cell morphology (Figure 1A). The breakdown of RPE (arrow, Figure 1B(i)) was observed after laser burn and an autofluorescent circular injury of BM [24] was also revealed (arrowhead). Confocal reflection microscopy demonstrates tissue fragments and cellular debris within the lesion (Figure 1B(ii)). In addition, TUNEL^+^ cells with damaged DNA were observed close to the lesion site immediately after laser (Figure 1B(iii)), whilst normal RPE/choroid from age-matched mice (6 to 8-week old) contained no apoptotic cells by TUNEL staining. There were no TUNEL^+^ cells found 2-7 days post laser (data not shown), demonstrating that laser induced cell apoptosis does not persist.

### Retinal microglia and blood monocytes rapidly accumulate and are activated at lesion sites prior to onset of angiogenesis

Given the rapid RPE damage and localised disruption in outer retinal barrier observed above, we wished to assess the temporal relationship between the formation of neovascular membrane and myeloid cell recruitment. Angiogenesis and neovascular membrane formation was assessed via immunostaining RPE/choroid whole-mounts with IB4 (Figure S1), which demonstrates that onset of choroidal angiogenesis begins on day 4 post laser, while formation of CNV membrane is established by day 7 and further developed between 7–14 days, prior to involution and consistent with previous reports [25]. The kinetics of macrophage recruitment to both RPE/choroid and retina was assessed via quantitative flow cytometric analysis of cell infiltrate. Overall, there is a significant increase of CD45^+^F4/80^+^ cells in RPE/choroidal tissue between day 1 and 7 as compared to control tissue (Figure 1C). The peak of infiltrate occurs on day 2 (490% increase vs. control) but cell numbers return to basal levels by day 14. In retina, elevated CD45^+^F4/80^+^ cell numbers were also observed after 2-4 days, with the maximal infiltrate on day 4 (120% increase) which is both later and less pronounced than the change in RPE/choroid. These results demonstrate that accumulation of macrophages to the site of injury is transient and early following initial laser damage and importantly occurs prior to the onset of choroidal angiogenesis.

Macrophage morphology indicates cell activation and differentiation [26]. To further examine local macrophage recruitment and activation, RPE/choroidal tissue was collected at different time points post laser and immunostained with Iba1, a marker for both microglia and monocytes (Figure 1D). Iba1^+^ staining confirms the transient pattern of cell accumulation between day 1 and 14, and demonstrates that these cells are localised within or adjacent to the site of RPE/BM injury. Furthermore, the recruited Iba1^+^ cells display different morphology. At day 1 post-laser (Figure 1D(ii)), ramified cells with small soma and long processes (white arrow) similar in appearance to retinal microglia (inset), as well as relatively round (yellow arrow) or amoeboid Iba1^+^ cells with enlarged cell body and shortened dendrites (red arrowhead) are all present. However, after 2 days (Figure 1D(iii)), fewer ramified microglia at lesions and a greater number of amoeboid cells are detected, which infers microglial activation [26].

As there is evidence of both activated microglia as well as potential infiltrating cells, we wished to confirm the involvement of microglia in the CNV progression. We first took advantage of the CX3CR1^*gfp/+*^ transgenic mouse in which resident microglia are fluorescently labelled. Confocal images (top and side views of Z-stacks) of retinas from CX3CR1^*gfp/+*^ mice were examined 2 days post laser, and clearly demonstrate migration of microglia from the inner retina toward the lesion site following laser application (Figure 1E). Secondly, utilising the distinct chemokine receptor expression differences between microglia and inflammatory monocytes, CCR2^-^CX3CR1^hi^ for the former and CCR2 ^+^ CX3CR1^lo^ for the latter [27,28], we noted that in RPE/choroidal and retinal whole-mounts from CX3CR1^*gfp/+*^ mice day 4 post laser both CCR2^-^ microglia-derived (arrow) and CCR2^+^ monocyte-derived (arrowhead) cells were present (Figure 1F). In support of CCR2^+^ cell infiltration we documented an increase in *Ccr2* mRNA in RPE/choroid (between day 1 and 4) and retina (day 4) (Figure 1G). Microglia do not express CCR2 [28], therefore the temporally enhanced *Ccr2* mRNA expression is likely due to the accumulation of blood-derived CCR2^+^ monocytes in response to CCL2 [25]. Furthermore, dissection of CNV lesions from whole RPE/choroid tissue on day 2 reveals greater increases in both *Ccl2* and *Ccr2* mRNA levels compared to peripheral RPE/choroid tissue (Figure 1H), supporting a focal chemokine response.

### Early and lesional-restricted inflammatory gene expression demonstrates a predominant Arg-1 expressing myeloid cell infiltrate

**Figure 2:**
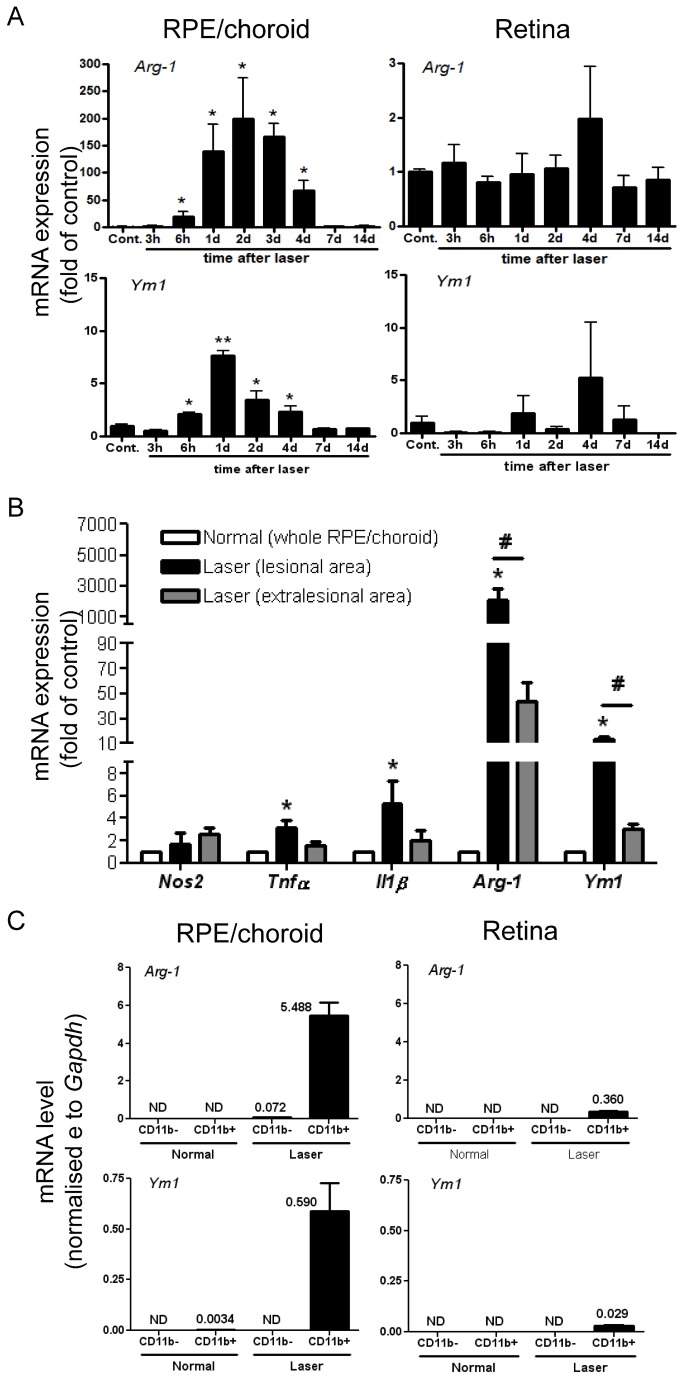
Early and local inflammation-associated gene expression is mainly from accumulating macrophages. (**A**) QRT-PCR analysis of time-dependent *Arg-1* and *Ym1* mRNA expression in RPE/choroid and retina tissues collected at indicated time points post laser induction. (**B**) RPE/choroid-lesional gene expression of *Nos2*, *Tnfα*, *Il1β*, *Arg-1* and *Ym1* from isolated lesions at day 2 confirms localised inflammatory gene signature. (**C**) Cellular gene expression of *Arg-1* and *Ym1* on day 2 using CD11b MACS-isolated cells pooled from 4 RPE/choroidal or retinal tissues demonstrates early and local *Arg-1* and *Ym1* expression largely produced by myeloid population. *Gapdh* served as a normalising control in (**A**–**C**). Data are presented as mean ± SEM, n=3-6 for each time point. ^*^ *P*<0.05 vs. control; ^**^ *P*<0.01 vs. control; ^#^ *P*<0.05 vs. non-lesion tissue on laser-treated eyes. ND, not detected.

Given the data above and the recognised immune activation and inflammation associated with development of angiogenesis related to AMD [3,8], we sought to characterize the phenotype of the accumulating macrophages and explore how this contributes to CNV development. Initially we examined mRNA expression to test whether a segregated M1 or M2 phenotype was present in the whole RPE/choroid and retinal tissues between 3 hours and 14 days post laser. In whole RPE/choroid, *Nos2* mRNA remained largely at basal level (Figure S2A). The expression of other M1 pro-inflammatory genes including *Il1β* and *Tnfα* were only slightly upregulated (<3 fold) in RPE/choroid during the early phase of CNV development (up to 7 days, Figure S2A) and we did not find significant changes of expression for *Il18*, *Il4* and *Il10* (data not shown). Notably, an M2 signature, *Arg-1* mRNA expression was increased after 6 hours, and further increased between day 1 and 4 (70-200 fold), and then returned to basal levels during the period 7-14 days (Figure 2A). Expression of another M2 gene *Ym1* demonstrated a similar pattern as *Arg-1*, although the induction fold was less (<8 fold, Figure 2A). Within retina, only *Tnfα*, showed any significant increase in gene expression during the early phase of CNV development (Figure S2A). In addition, mRNA level for *Cd200 receptor* (*Cd200r*), a myeloid-specific inhibitory receptor [29], was increased after 4-7 days in RPE/choroidal or retinal tissue (Figure S2A), which is generally later than other genes we tested.

When lesions were isolated from the whole RPE/choroid on day 2 post laser the signature of *Arg-1* was enhanced (Figure 2B) and to a lesser extent *Tnfα*, *Il1β*, and *Ym1* transcripts were increased. To determine if the *Arg-1* signature resided in infiltrating myeloid cells, we isolated CD11b^+^ cells from pooled single-cell suspensions of RPE/choroid or retina. In normal retina, there was negligible signal in either CD11b^-^ or CD11b^+^ (microglia) cells (Figure 2C). Conversely two days post laser, mRNA expression for *Arg-1, Ym1, Tnfα* and *Il1β* was largely restricted to CD11b^+^ cells from RPE/choroid (Figures 2C and S2B). The expression of Arg-1 by early accumulating myeloid cells was further confirmed by conventional RT-PCR (Figure S3A) and immunostaining showing cytoplasmic expression of Arg-1 in lesional CD11b^+^ cells on RPE/choroid whole-mounts collected on day 3 (Figure S3B).

### Accumulating microglia/myeloid cells supply the earliest enhancement of VEGF expression within the lesion

Whilst we have described a specific macrophage phenotype prior to angiogenesis and although VEGF expression by macrophages [30–32], as well as other cells including RPE and neovascular endothelial cells [30,33] are described in experimental CNV, the chronological order of VEGF expression in these different cell types during CNV development or what initiates the drive toward pathological angiogenesis following injury is unknown.

**Figure 3:**
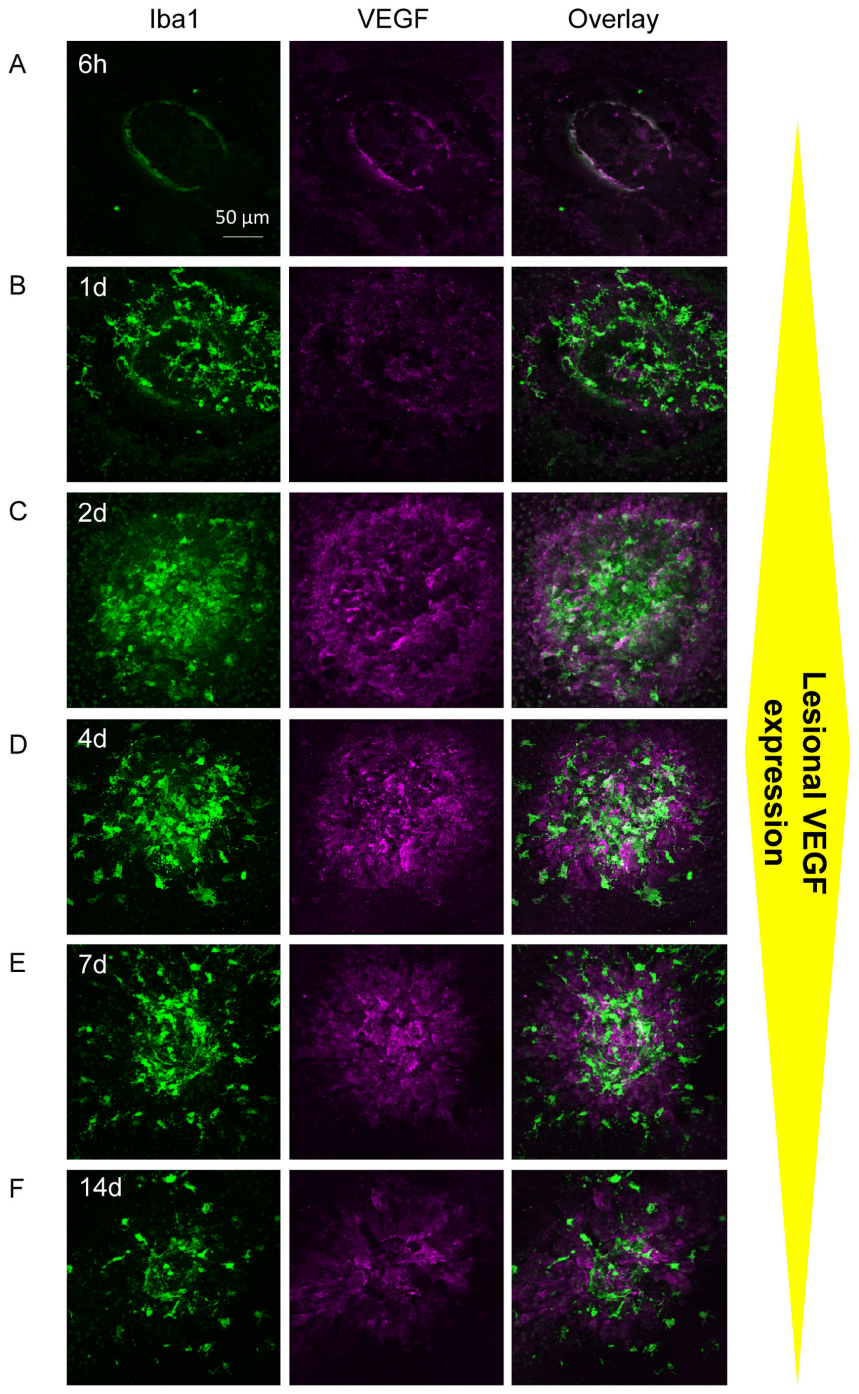
Lesional VEGF expression in laser CNV model. Iba1 and VEGF dual-staining and confocal microscopy on RPE/choroid whole-mounts display transient VEGF expression at lesion site. (**A**) 6 hours post laser, no increase of Iba1^+^ cells or VEGF immuno-reactivity was found at laser lesions. (**B**) On day 1, recruited ramified microglia and amoeboid macrophages express VEGF (also see Figures S4 and S5). By day 2 (C) and 4 (D), greater VEGF immuno-reactivity are found in accumulating macrophages, as well as in Iba1^-^ cells. Between day 7 (E) and 14 (F), lesional VEGF expression lessened, in parallel with reduced macrophage number. Images are representatives of 12-24 lesions for each time point.

In this study, a transient VEGF expression by accumulating macrophages during early stages (days 1-4) of the laser-CNV model was detected using immunostaining and confocal microscopy (Figure S4), as described in primates [30]. Within the normal tissue, resting microglia distributed sparsely in the sub-retinal space have no significant VEGF immuno-reactivity (Figure S4A). Six hours after laser induction, no significant Iba1^+^ cells and VEGF upregulation were observed within the lesion that contains abundant damaged cells and debris. By day 1 the majority of VEGF immuno-reactivity was clearly co-localised to recruited microglia or myeloid cells (Figure 3B). This is further confirmed when analysing the fluorescence plot profiles which shows that the VEGF signal peaks in Iba1^+^ cells and only low levels in RPE adjacent to the lesion on day 1 (Figure S5), the time when myeloid cells accumulate. This indicates that the earliest increase in VEGF within the lesion is produced by recruited microglia/myeloid cells. By day 2, the majority of lesional Iba1^+^ cells were co-localised with enhanced VEGF expression (Figure 3C). At this time point increased VEGF expression by the surrounding non-myeloid cells was also observed. VEGF expression in Iba1^+^ cells was maintained until day 4 (Figure 3D). Between day 7 and 14 post laser, lesional VEGF immuno-reactivity subsided, coinciding with the reduced number of VEGF^+^ macrophages (Figure 3E,F).

### Recruited myeloid cells are largely non-proliferating and their accumulation precedes recruitment of proliferating non-myeloid cells

**Figure 4:**
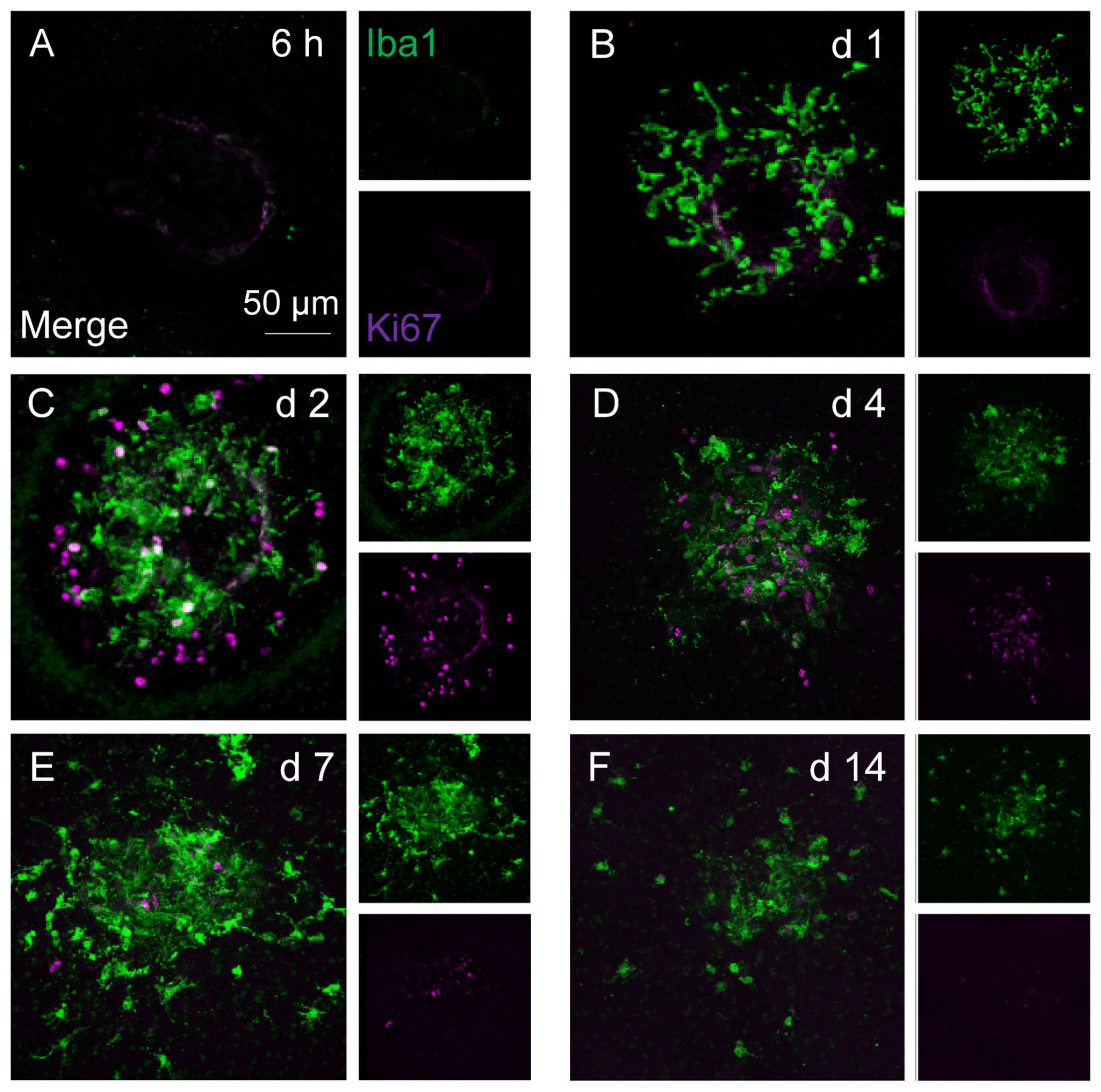
Microglia/monocyte accumulation precedes appearance of non-myeloid cell proliferation. Iba1 and Ki67 dual-staining on RPE/choroid whole-mounts during the time course of CNV development. (**A**) Six hours post laser induction, neither macrophages nor cell proliferation are detected at lesions. (**B**) On day 1, accumulating Iba1^+^ cells are Ki67-negative and no proliferating cells are observed. By day 2 (C) and 4 (D), majority of macrophages are not co-localised with Ki67 immuno-reactivity, while abundant Iba1^-^Ki67^+^ cells are seen at lesions. Between day 7 (E) and 14 (F), both Iba1^+^ and Ki67^+^ cell numbers subside. Images are representative of at least 12 lesions for each time point. Smaller images display separate stains for Iba1 and Ki67, respectively; bigger images are two channels merged.

To interrogate whether *in situ* myeloid cell expansion at the lesion site contributes to increased number of myeloid cells or to endothelial proliferation we co-immunostained Iba1 with Ki67. As shown in Figure 4A, neither myeloid nor proliferating cells were detected at lesions 6 hours post laser. On day 1 (Figure 4B), accumulating microglia/monocytes showed no proliferative capacity and there were no Ki67^+^ cells observed. A number of Ki67^+^ cells were seen at lesions on days 2 and 4, although largely not Iba1-positive (Figure 4C,D). Between day 7 and 14, Ki67^+^ cell expression subsided (Figure 4E,F). The data demonstrate that cell proliferation does not contribute to myeloid cell numbers observed and the accumulation of lesional myeloid cells occurs prior to notable non-myeloid proliferation or morphological evidence of angiogenesis. We did not pursue whether Ki67^+^ cells were endothelial or endothelial progenitors [33].

### Microglia recruitment and VEGF expression persist after depletion of CCR2^+^ monocyte infiltrate

**Figure 5:**
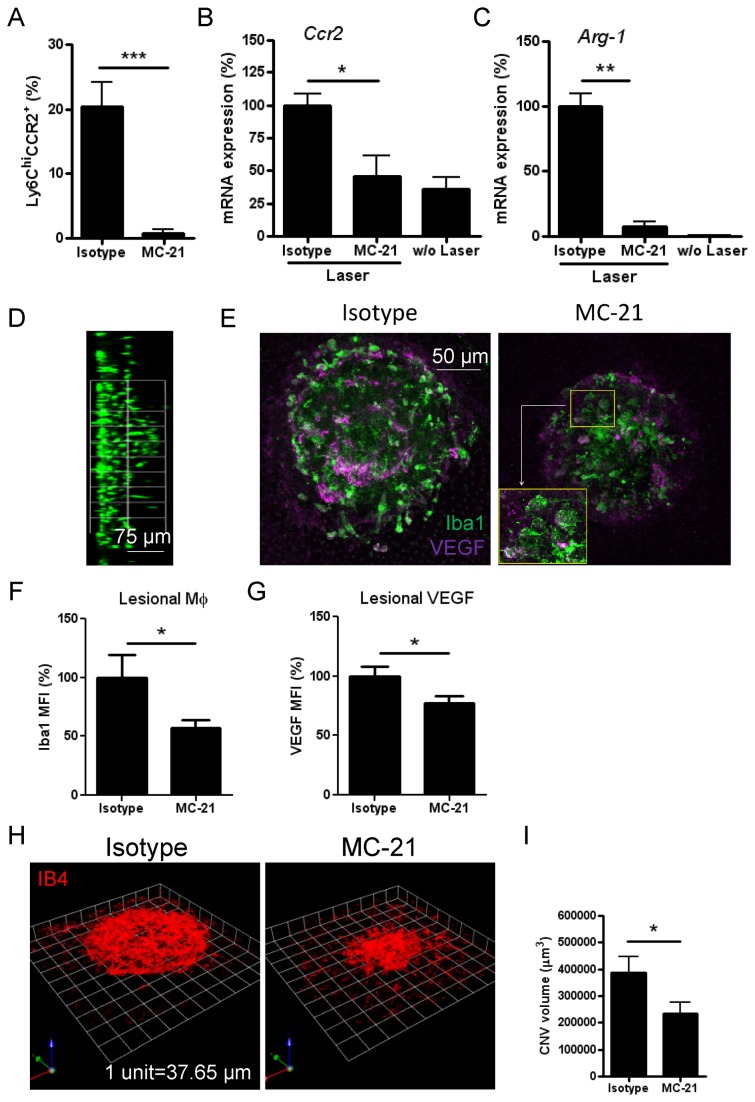
Systemic depletion of CCR2^+^ monocytes abolishes *Arg-1* signature without altering microglia migration, and partly suppresses laser-induced CNV formation. (**A**) Flow cytometric analysis of peripheral blood from mice following depletion of Ly6C ^hi^CCR2^+^ monocyte subset using the MC-21 anti-CCR2 mAb, compared with rat IgG2b isotype control (mean ± SEM, n=3). Assessment of *Ccr2* (**B**) or *Arg-1* (**C**) mRNA expression normalised against *Gapdh* in RPE/choroid day 2 post laser in MC-21 treated mice by QRT-PCR (mean ± SEM, n=4-6). (**D**) Side view of Z-stacks of confocal images shows migration of retinal microglia (Iba1 staining) in MC-21 treated animals. Confocal images (**E**) and semi-quantitative mean fluorescence intensity (MFI) analysis using ImageJ software for Iba1^+^ cell accumulation (**F**) and whole lesional VEGF expression (**G**) on RPE/choroid whole-mounts taken on day 2 post laser (mean ± SEM, n≥12/group). Magnification image in (E, inset) shows macrophages co-stained for VEGF in MC-21 injected mice. (**H** and **I**) CNV formation on day 7 post laser was assessed by IB4 staining on RPE/choroidal whole-mounts and images were captured by confocal microscopy and analysed using a Volocity® 3D Image analysis software. Representative 3D reconstructions of neovascular complex (**H**) and quantitative data (I) show reduced CNV volume by 39% with MC-21 treatment compared with control (n=24/group). ^*^ *P*<0.05; ^**^ *P*<0.01; ^***^ *P*<0.001.

Our data thus far demonstrate that laser-induced injury at RPE/BM causes rapid macrophage recruitment from retina (CCR2^-^ microglia) and blood (CCR2^+^ monocytes), leading to early and local Arg-1 and VEGF expression, prior to formation of neovascular membranes. To further determine the role and extent of blood monocytes, we depleted CCR2^+^ cells with an anti-CCR2 (MC-21) mAb, which selectively depletes Ly6C ^hi^CCR2^+^ monocytes from peripheral blood [34–36]. Following a daily i.p. injection regimen of MC-21, blood Ly6C ^hi^CCR2^+^ monocytes were depleted by 92% on day 2 post laser (Figure 5A). Depletion resulted in suppression of *Ccr2* mRNA induction (Figure 5B), loss of CCR2 immuno-positive cells (Figure S6), and reduced *Arg-1* mRNA expression by 90% (Figure 5C) in RPE/choroid. However, migration of microglia to the lesions was not affected by CCR2^+^ monocyte depletion (Figure 5D). Loss of *Ccr2* and *Arg-1* signature also corresponded to reduced Iba1 immuno-reactivity at the lesion site (Figure 5E,F). The 40% reduction observed represents the percentage of infiltrating monocytes among the total number of myeloid cell infiltrate. Similarly lesional VEGF immuno-reactivity was downregulated by MC-21-mediated CCR2 monocyte depletion in laser-treated animals (Figure 5E,G). However, microglia at site of injury in CCR2-depleted mice remained VEGF expressing (Figure 5E and inset). Although microglia recruited to the lesion site remain functional in generating VEGF, MC-21 administration suppressed angiogenesis by 39% on day 7 post laser induction (Figure 5H,I), confirming that depletion of CCR2^+^ monocyte infiltration suppresses angiogenic development.

### Myeloid cell uptake of damaged RPE or its derivatives conditions toward Arg-1 phenotype and VEGF production

**Figure 6:**
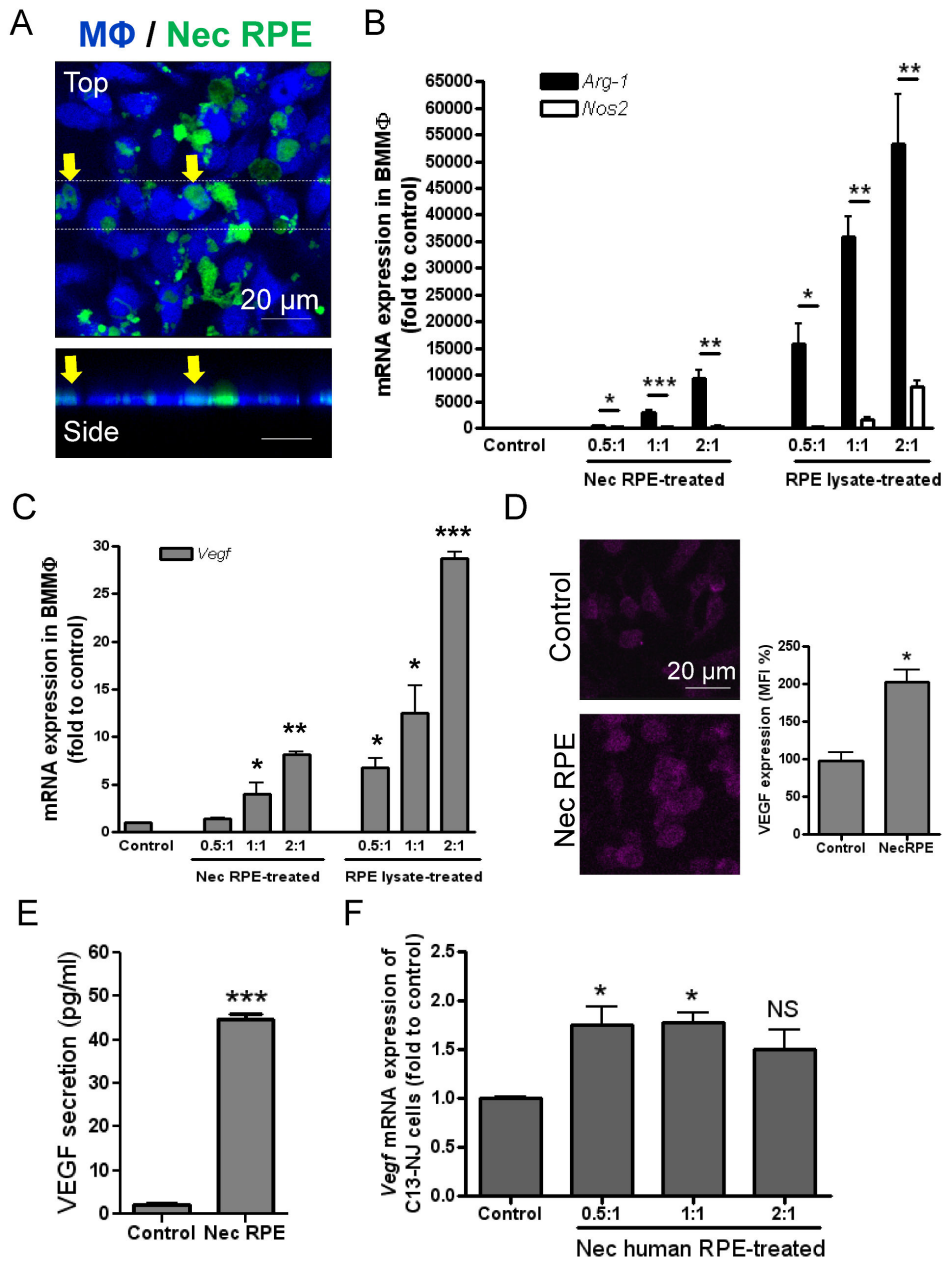
Macrophage uptake of necrotic RPE or its derivatives altering their inflammatory and proangiogenic phenotype. (**A**) Necrotic RPE was produced from CFDA-labelled B6-RPE07 cells (green) by heating at 95°C for 15 minutes, while RPE lysate was generated by sonication for 5 minutes on ice. Necrotic RPE cells or lysate were then added to Violet Tracer-labelled BMMΦs at different ratios (RPE to BMMΦ). After 1 hour incubation, the cells were washed, fixed and observed by confocal microscopy. Top and side view of confocal images shows engulfment of dead RPEs/debris by BMMΦs. BMMΦs were collected after 24 hours of incubation with necrotic RPE or cell lysate and RNA was extracted for QRT-PCR analysis to determine *Arg-1* and *Nos2* (**B**), and *Vegf* gene expression (**C**). *18s rRNA* was used as a normalising control. (**D**) Confocal images and MFI analysis of VEGF immunocytochemistry on BMMΦs treated with necrotic RPE. (**E**) In some of experiments, after 1 hour of incubation of BMMΦs with necrotic RPE, un-engulfed dead RPE cells and its derivatives were removed and BMMΦs were further cultured for 24 hours and cell culture supernatants collected for determination of VEGF concentration using ELISA. (**F**) Human microglia cells (C13-NJ cell line) were treated with heat-induced necrotic ARPE19 cells at different ratios for 24 hours and then examined for *Vegf* mRNA expression. Data are presented as mean ± SEM, n≥3. ^*^ *P*<0.05; ^**^ *P*<0.01; ^***^ *P*<0.001 *Arg-1* vs. *Nos2* (**B**), or vs. control. NS, not significant.

Iba1 immunostaining demonstrates macrophage surface ruffling on cells within the lesion (Figure S7A, arrow), a membrane change associated with cell phagocytic activity [37]. Furthermore, after laser application, fragmented RPE pigments were observed within the lesion and on both RPE/choroid and outer retina aspects. Despite abundance of RPE pigments we could elucidate pigment-engulfing macrophages at the lesion site from the retina side (Figure S7B), where brightfield images were colour-processed from black(pigment)/white(retina) to green(pigment)/black(retina) and then merged with Iba-1 staining (red). A pHrodo Red-Dextran endocytosis assay based on *ex vivo* retina culture also confirmed endocytic activity of lesional macrophages (Figure S7C). Based on this data, and to test whether uptake of damaged RPE/derivatives by lesional macrophages induced the phenotype and function described above, we adapted an *in vitro* phagocytosis assay in which heat-induced necrotic mouse RPEs (B6-RPE07) were added to bone marrow-derived macrophages (BMMΦs). Engulfment of dead RPEs and debris by macrophages was observed after 60 minutes (Figure 6A) and demonstrated dose-dependent induced BMMΦ *Arg-1* mRNA expression (Figure 6B). *Nos2* mRNA expression in macrophages conditioned by necrotic RPE or cell lysate is significantly less than *Arg-1*, which is generally consistent with our *in vivo* finding. In addition, macrophage VEGF expression at both transcript and protein levels were significantly enhanced by treatment with necrotic RPE or lysate (Figure 6C–E). To confirm our *in vivo* finding that microglia-derived macrophages also express VEGF, we analysed *Vegf* gene expression in a human microglia cell line (C13-NJ) treated with necrotic human RPE cells (ARPE-19). As expected, *Vegf* mRNA level in the human microglia cells was enhanced by addition of dead RPE cells (Figure 6F).

We then tested whether oxidative stress-induced RPE apoptosis could result in similar macrophage responses as necrotic RPE. H_2_O_2_ treatment of B6-RPE07 cells induces apoptosis as analysed by annexin V/7AAD staining (Figure S8A), and the *in vitro* phagocytosis assay demonstrates engulfment of apoptotic RPE/debris by BMMΦs within 60 minutes (Figure S8B). When BMMΦs were subsequently isolated using CD11b microbeads from the phagocytic assay co-culture after 24 hours of incubation (>95% purity analysed by flow cytometry), apoptotic RPEs induced a dose-dependent upregulation of macrophage *Arg-1* as well as increase in *Nos2* mRNA from BMMΦs (Figure S8C), and enhanced *Vegf* expression (Figure S8D).

**Figure 7:**
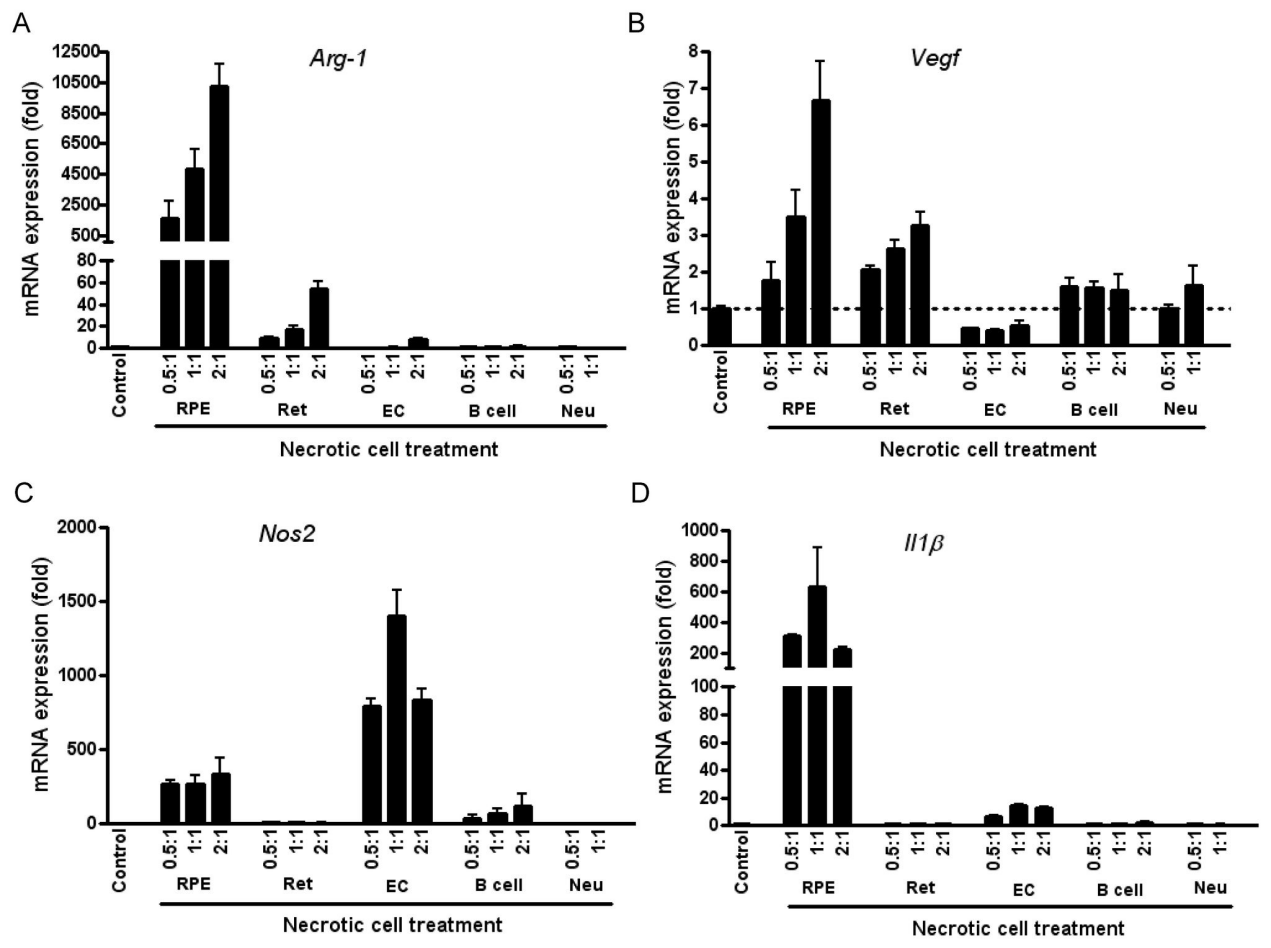
Gene expression of BMMΦs in response to different necrotic cell types. Mice BMMΦs were treated with necrotic cells generated from B6-RPE07 cells (RPE), whole retinal tissues (Ret), retinal vascular endothelial cells (EC), YTS156 hybridoma (B cell) or bone marrow neutrophils for 24 hours. Cellular RNA was isolated and gene expression of *Arg-1* (**A**), *Vegf* (**B**), *Nos2* (**C**) and *Il1β* (**D**) was assessed by QRT-PCR (mean ± SEM, n≥3). 18s rRNA was utilised as a normalising control and results presented as fold change of control.

Employing other necrotic cell sources including retinal cells (excluding RPE), retinal endothelial cells, B-cell hybridoma (YTS156) and bone marrow neutrophils did not confer the robust *Arg-1* phenotype in BMMΦs we observed with necrotic RPE feeding (Figure 7). For example, whilst uptake of necrotic retinal endothelial cells promoted macrophage *Nos2* expression there was no upregulation of *Vegf* or *Arg-1* (Figure 7A–D). Furthermore, co-culture with necrotic BMMΦs did not elicit a macrophage response. The *in vitro* data support the contention that early macrophage *Arg-1* phenotype and proangiogenic response (VEGF expression and secretion) is induced by damaged RPE.

## Discussion

Central to retinal degenerative diseases, including AMD, is dysregulation of control mechanisms that help maintain normal tissue homeostasis and integrity [9]. Medzhitov has proposed para-inflammation as a tissue-adaptive response to noxious stress or malfunction, the physiological purpose of which is an attempt to re-establish tissue homeostasis [7]. However, if homeostasis is not redressed, the mechanisms evoked may become persistent and paradoxically promote chronic inflammation, resulting in non-healing wounds and ongoing tissue damage [8,9,38,39]. Whilst angiogenesis is critical to the process of tissue repair, in the ageing retina, pathological conditions leading to initial retinal damage become persistent, and uncontrolled and pathological angiogenesis may develop in the subretinal space resulting in severe retinal impairment [23,40].

Whilst extensive clinical evidence implicates accumulation and activation of macrophages in the subretinal space during pathogenesis of AMD [3,20], whether these cells provide the drive toward angiogenesis has not previously been demonstrated. The data presented in this study identify the earliest event following disruption of RPE/BM with laser is a heterogeneous myeloid cell infiltrate from both retina (microglia) and infiltrating CCR2^+^ monocytes at the same time as considerable tissue, and in particular RPE cell death. Furthermore, the recruited myeloid infiltrate expresses a definitive gene signature of selective inflammatory mediators together with pronounced Arg-1 and VEGF expression, and importantly this is prior to any pathological signs of disease. These findings raise important questions as to whether incoming macrophages have a sentinel role or act to clear the cellular debris, or their presence indicates a central and initial drive toward angiogenesis. Several novel features are realised from the current data in support of the latter hypothesis.

Firstly, the rapid increase of myeloid cell numbers in the initial few days, prior to any morphological evidence of angiogenesis includes populations of both CCR2-positive (monocytes) and CX3CR1-positive (microglia) cells. To date, clinico-pathological identification of the source of recruited macrophages has proven technically difficult [1,3], although this is a critical issue relevant to any immune-modulating treatment for the disease. Studies using CCL2- or CCR2-deficient animals have proposed infiltrate of inflammatory monocytes (CCR2^+^) in experimental CNV models, which is further supported by the elevated intraocular CCL2 level in patients with neovascular AMD [41]. The contribution of microglia, the tissue resident macrophages of the central nervous system and neuroretina, which are essential for tissue homeostasis and innate immune defence must also be considered [9]. Indeed, dysregulated microglia activation is recognised in chronic inflammation-induced neurodegenerative disorders in the central nervous system, such as Parkinson’s disease, Alzheimer’s disease and multiple sclerosis [42]. Thus evidence that microglia both accumulate in the laser injury model, and upregulate their VEGF expression in response to necrotic RPE, strongly suggests the microglia also contribute to angiogenic development.

Local upregulation of *Ccl2* and *Ccr2* gene expression within the lesion supports the concept that the infiltration of peripheral monocytes is CCL2/CCR2-mediated [17]. However no observed change in *Cx3cl1* expression suggests that migration of microglia to the lesion may be CX3CL1-independent, and further studies will be required to delineate the molecular mechanisms underlying RPE/BM injury induced motility response. Regardless of the likely distinct mechanisms for monocyte and microglia accumulation, both myeloid types are rapidly activated at the site of injury, as evidenced by changes from their original phenotype to amoeboid morphology. Nevertheless, it was not possible to highlight the exact contribution of myeloid cell subtypes during CNV evolution in our system because of the likely dynamic change in cell phenotype. As presented we show at early stages there are largely two populations of myeloid cells, CCR2 ^+^ CX3CR1^lo^ and CCR2^-^CX3CR1^+^, but we also noted that there were dual-positive cells in various proportions (data not shown).

Secondly, as shown previously [25] and in this study (Figure S9), although complement is activated and the membrane attack complex (MAC) deposited within the laser lesion in rapid response to the insult (within 3-6 hours), there is little loss of cells adjacent to the injured site as demonstrated by TUNEL assay and such early complement deposition does not result in contemporaneous upregulation of RPE-VEGF expression until after microglia/myeloid cell accumulation. Cytokines produced from microglia/myeloid cells, such as IL1β and TNFα, have been previously shown to enhance expression of complement factor B from RPE, and thus stimulate the alternative complement pathway related to AMD pathogenesis [43,44]. Indeed, our results show that the recruited microglia/monocytes lead to further complement activation as evidenced by massive MAC deposition on day 1. In parallel with decreased macrophage accumulation and cytokine gene expression after 7-14 days, MAC expression also reduced (Figure S9).

Thirdly, we observed that the recruited macrophages are conditioned toward a consistent signature of an Arg-1 phenotype within the lesion. Although previous studies have demonstrated the involvement of macrophages in CNV, this is without clarification of cell phenotype and function [20]. It is recognised that M2 macrophages are associated with pathological angiogenesis [11], as well as in experimental models of retinopathy, such as retinal ischemia and chronic stages of autoimmune uveoretinitis [13,45]. Here, we demonstrate that macrophages recruited following RPE/BM injury are responsible for an early (up to 4 days post laser) local inflammatory response. The recruited lesional macrophages exhibit to a lesser extent gene expression of M1 markers, including *Il1β*, *Tnfα* and *Nos2*, and a greater bias toward induction of M2 markers, particularly *Arg-1*. Isolated CD11b^+^ cells from RPE/choroid on day 2 post laser have much stronger *Arg-1* mRNA level than CD11b^-^ cell populations, demonstrating that the early recruited myeloid cells are the main source of Arg-1 at this time point, although surrounding RPE cells may contribute to the overall arginase (isoforms 1 and 2) production at a latter stage during the disease progression [46]. Arg-1 has an important role in regulating inflammation and is involved in chronic inflammatory conditions and vascular dysfunction in retinopathy [11,47,48]. Arg-1 catalyses the conversion of arginine to ornithine, a precursor of polyamines and collagen, thus contributing to production of extracellular matrix that supports angiogenesis [48]. Thus, Arg-1 may not only be a phenotypic signature of VEGF secretion from myeloid cells, but also actively function as a mediator during angiogenic development. The increased *Cd200r* expression in both RPE/choroid and retina 4-7 days post laser suggests that the macrophages may undergo a CD200R-mediated inhibition at later stages, in agreement with reduced cell accumulation and inflammatory gene expression.

VEGF expression by infiltrating macrophages in the mouse laser-CNV model, and the prominent role of VEGF in this context is recognized [25,31,32]. However, recent data demonstrates VEGF expression in the nuclei of surrounding RPE cells of laser lesions at 66 hours and 5 days post-laser in VEGF reporter mice (*Vegfa* ^*lacZK/WT*^), but not in F4/80^+^ macrophages or SMA^+^ myofibroblasts [49]. Furthermore, targeted deletion of macrophage or RPE derived VEGF expression does not prevent CNV formation or alter CNV size, indicating that VEGF-independent mechanism(s) also exist to initiate choroidal angiogenesis. Our time-course studies provide evidence that rapidly recruited microglia/myeloid cells are the earliest source for VEGF production within the lesion at day 1, and its expression further increases over days 2 and 4, but is then lost between day 7 and 14. This highlights both the early and transient contribution of myeloid cell derived VEGF to CNV evolution.

Our current data, whilst indirect, supports VEGF expression in both CCR2^+^ monocytes and CCR2^-^ microglia at the injury site. Firstly activated microglia and myeloid cells are recruited to site and show Iba1^+^ and amoeboid morphology (Figure 1), and secondly, Iba1 and VEGF expression co-stain (Figure 3, S4 and S5). Lastly, depletion of the CCR2^+^ infiltrate still results in VEGF expression in Iba1^+^ cells, likely derived from microglia. VEGF provides a crucial signal which attracts bone marrow-derived endothelial progenitor cells that in turn proliferate to form new blood vessels [33], and importantly our results highlight that recruitment of VEGF-positive macrophages occurs before any overt signs of cell proliferation at the lesion site. A recent publication does describe proliferation of some myeloid cells at lesions on day 3 post CNV induction that contributes to the myofibroblast scaffold, supporting angiogenesis [49]. In the current study, whilst majority of infiltrating myeloid cells were not proliferating, Iba1-negative populations did proliferate, which may represent differentiated myeloid or endothelial precursor cells.

In addition to generating proangiogenic factors such as VEGF, macrophages also act as bridging cells between endothelial tip cells permitting fusion and the formation of new continuous blood vessels [50]. In support of other data demonstrating that macrophages can promote RPE-VEGF expression via macrophage-derived TNFα and IL1β [51], enhanced *Tnfα* and *Il1β* mRNA expression from these early recruited macrophages further emphasize the importance of macrophages in promoting VEGF expression from surrounding RPE and mediate choroidal angiogenesis. It should be noted that expression of VEGF from both the accumulating macrophages, and surrounding RPE, subsides by the time new vessels are formed, suggesting that a key functional role of macrophages is to initiate as opposed to previously held notions that they maintain CNV.

Importantly, depletion of CCR2-positive cells results in loss of *Arg-1* signature, demonstrating that *Arg-1* expression resides mainly in infiltrating monocytes. This finding indicates a possible role of monocytes-derived macrophages in regulation of laser-induced inflammation as observed in a murine model of glutamate eye intoxication [52]. Furthermore, we show that MC-21 administration suppresses angiogenesis by 39% on day 7 post laser induction (Figure 5I), confirming that depletion of CCR2^+^ monocyte infiltration inhibits angiogenic development, although microglia recruited to the lesion site remain functional, generating VEGF and contributing toward CNV progression. The mechanisms that control retinal microglia migration to laser injury sites remain unclear. Recent *in vitro* evidence demonstrates that extracellular ATP-induced ATP release from microglia lysosomes is critical in mediating microglia migration to injured tissue [53]. Such pathways may provide potential targets that may experimentally facilitate inhibition of microglia accumulation during the early stage of the disease progression, thus confirming the full extent and contribution of microglia to CNV formation.

Fourthly, our data show that myeloid cells are actively phagocytic both *in vivo* and *in vitro* and it is largely the uptake of damaged RPE or its derivatives that induces the Arg-1 and VEGF expression. In general, and potentially operative in this model, the process of cell necrosis is likely to be initiated by exposure to toxic substances, hypoxia and/or excessive inflammation [48], which in turn may drive proangiogenic macrophage conditioning. In relation to AMD, RPE death and dysfunction have been observed during different stages of the disease [1–3]. *In vitro* studies have shown that macrophages phagocytose necrotic tumour cells through a phosphatidylserine-dependent pathway without production of pro-inflammatory cytokines [54]. In addition, cell component degradation in phagosomes is essential to activate macrophage inflammasome [55]. Our demonstration that necrotic cell preparation from retinal vascular endothelial cells induces significant *Nos2* gene expression, but not *Arg-1*, suggests that dysfunction at the level of retinal endothelium may evoke inflammation in the eye, akin to findings of endothelial dysfunction along with retinal inflammation in diabetic retinopathy [56]. RPE apoptosis has been recognised as an important step at the initial stage of retinal degeneration and ocular neovascularisation [57]. Our results demonstrate that H_2_O_2_-induced RPE apoptosis stimulates both *Nos2* and *Arg-1* expression in macrophages, thus confirming that oxidative stress-mediated apoptosis generates a chronic inflammatory and toxic environment in ageing eyes [2,3]. Notably, macrophage *Vegf* expression was upregulated by either apoptotic or necrotic RPE, suggesting that RPE damage, regardless of pathological causes leading to the damage, conditions macrophages toward a proangiogenic state thus promoting CNV.

It is known that both heat stress and oxidative stress can lead to oxidative damage in cellular contents [58]. Healthy RPE is highly active in metabolising lipid-rich photoreceptor outer segments through lysosomal digestion of phospholipids. However, dysfunctional RPE in ageing eyes reduces the lysosomal efficiency, resulting in accumulation of incompletely digested and oxidised lipoproteins, recently identified as low density lipoprotein (oxLDL) [59]. Uptake of oxLDL by macrophages *in vitro* can induce Arg-1 and VEGF production [60,61], which may provide an explanation for the highly specific effect of damaged RPE to regulate macrophage phenotype we observed in this study. It is recently reported that impaired cholesterol efflux in senescent macrophages with downregulated ABCA1 causes elevated intracellular lipid levels, which generates an M2 phenotype and promotes vascular proliferation in CNV models [5]. These data maintain support for a critical role of abnormal lipid metabolism in conditioning and determining myeloid cell function as seen during the initiation of angiogenesis related to AMD and supported by the observation of early myeloid cell infiltration prior to lesion development in CEP-induced model of AMD [62].

In conclusion, our results demonstrate that laser injury induces an early inflammatory response prior to development of choroidal angiogenesis, which involves recruitment of heterogeneous macrophages derived from monocytes and microglia. Phagocytosis of damaged RPE components conditions myeloid cells to adopt an Arg-1 ^+^ VEGF^+^ M2-like phenotype, a previously unrecognised early mechanism underlying the initiation of choroidal angiogenesis. The data suggest that suppression of microglia mobility, myeloid activation or protection against RPE death may be beneficial for prevention of these early events that trigger CNV.

## Materials and Methods

### Mice and CNV induction

C57BL/6J mice (no *Rd8* mutation detected) were obtained from the Jackson Laboratory and breeding colonies were established within the Animal Services Unit at University of Bristol, UK. Mice were kept in the animal house facilities of the University of Bristol, according to the Home Office Regulations. CX3CR1^*gfp/+*^ mice (C57BL/6 background, Jackson Laboratory), in which one allele of *Cx3cr1* gene is replaced by *Egfp* gene [63], were bred and maintained in the Biological Resource Unit of Queen’s University, Belfast. Treatment of the animals conformed to the Association for Research in Vision and Ophthalmology (ARVO) statement. The experiments were conducted following protocols of the Home Office Project Licence 30/2745, approved by the University of Bristol Ethical Review Group.

CNV was induced by laser photocoagulation in mice aged 6-8 weeks. In brief, the pupils of animals were dilated using topical 1% tropicamide and mice anesthetized by intraperitoneal (i.p.) injection of 200 µl of Vetelar and Rompun mixed with sterile water in the ratio 0.6:1:84. Four laser spots were delivered to the posterior retina using an OculightSlx Krypton Red Laser system (power 200 mW, duration 75 ms, spot size 75 µm), unless in macrophage enrichment experiments where 8 laser burns were induced to obtain larger cell numbers.

### Antibodies

APC-conjugated rat anti-mouse CD45 (30-F11,1: 100), Alexa Fluor 488 or biotin-conjugated rat anti-mouse CD11b (M1/70, 1:100), and fluorochrome-isotype antibodies were purchased from BD Biosciences. Alexa Fluor 647-labelled rat anti-mouse/rat Ki-67 (SolA15, 1:100) was from eBioscience. Rabbit polyclonal anti-Iba1 (1:500) was obtained from Wako Chemicals. Rabbit polyclonal anti-Arg-1 (1:50) was from Santa Cruz Biotechnology. Chicken polyclonal anti-VEGF (1:100), DyLight 650-goat anti-chicken IgY (1:400) and goat polyclonal anti-CCR2 (1:100) were from Abcam. Rabbit polyclonal anti-C9 (1:500) was generated at Professor B. P. Morgan’s laboratory in Cardiff University. Rabbit polyclonal anti-Claudin (1:100), Alexa Fluor 488 or 568-conjugated goat anti-rabbit IgG, Alexa Fluor 488-conjugated rabbit anti-goat IgG (1:400), and PE-labelled rat anti-mouse F4/80 (BM8, 1:100) were obtained from Life Technologies. DL557-donkey anti-goat IgG (1:200) was from R&D Systems.

### RPE/Choroidal and retinal whole-mounts and confocal microscopy

Eyes were enucleated and fixed in 2% (wt/vol) paraformaldehyde (PFA). After dissection of retina and RPE/choroid, tissues were blocked and permeabilized in 5% BSA, 5% goat or donkey (for CCR2 staining only) serum with 0.3% Triton X-100 in PBS for 2 hours, followed by incubation with primary antibodies in 1% BSA with 0.15% Triton X-100 at 4°C overnight or for 2 hours (for Arg-1 staining). After thorough washing, samples were further incubated with secondary antibodies at RT for 3 hours in the dark. Tissues were washed and flat-mounted in Vectashield antifade medium (Vector Laboratories) and examined using a Leica TCS-SP2-AOBS confocal laser scanning microscope. Isotype antibodies or negative controls with primary antibody omitted did not show significant fluorescence signal. For evaluation of neovascular membrane on RPE/choroid, fixed tissues were blocked with 1% BSA and 0.5% Triton X-100, followed by incubation with biotin-conjugated isolectin B4 (IB4, Sigma-Aldrich) in PBS containing 1% Triton X-100, 0.1mM CaCl_2_ and 0.1mM MgCl_2_ overnight. Samples were washed before incubation with Rhodamine Red-X-labelled streptavidin (Jackson ImmunoResearch Laboratories) for 2 hours, and whole-mounts were prepared for confocal microscopy. The volume of choroidal neovascular complex was measured using a series of Z-stack images (from the surface to the deepest focal plane) using the Volocity® Image Analysis Software 6.0. TMR Red-dUTP TUNEL (Roche) staining of DNA breaks was performed on RPE/choroidal whole-mounts according to previous reports [64].

### Quantitative RT-PCR (QRT-PCR)

Total RNA from tissues or cell cultures was isolated using TRIzol reagent (Life Technologies). One µg of total RNA was treated with RQ1 RNase-free DNase before reverse-transcription using the ImProm-II^TM^ Reverse Transcription System (Promega). cDNA was amplified using the Power SYBR® Green PCR Master Mix Reagent (Applied Biosystems) on a StepOne™ Real-Time PCR System (Applied Biosystems). Primer sequences for mouse *Gapdh*, *18s rRNA*, *Arg-1*, *Tnfα* and *Vegf* were described previously [12,65]. Primers for other mouse or human genes were designed using the PrimerQuest online tool (http://eu.idtdna.com/Scitools/Applications/Primerquest/): mouse *Ccr2*, forward 5’-AAT GAG AAG AAG AGG CAC AGG GCT, reverse 5’-ATG GCC TGG TCT AAG TGC TTG TCA; mouse *Ym1*, forward 5’-TCA CAG GTC TGG CAA TTC TTC TG, reverse 5’-TGC ATT CCA GCA AAG GCA TA; mouse *Cd200r*, forward 5’-TGT GGC TGG GTC AAG TTG TAC TGA, reverse 5’-AAG CAG CAG AGC AGA GCC TTT GTA; mouse *Nos2*, forward 5’- TGG TCC GCA AGA GAG TGC T, reverse 5’- CCT CAT TGG CCA GCT GCT T; mouse *Il1β*, forward 5’-GCC CAT CCT CTG TGA CTC AT, reverse 5’-AGG CCA CAG GTA TTT TGT CG; human *18s rRNA*, forward 5'-CAC GGA CAG GAT TGA CAG ATT, reverse 5'-GCC AGAGTCTCGTTCGTTATC; human *Vegf*, forward 5'-TGG TGT CTT CAC TGG ATG TAT TT, reverse 5'-AGT CTC TCA TCT CCT CCT CTT C.

### CCR2^+^ monocyte depletion

MC-21(rat anti-mouse CCR2) or isotype control (IgG2b) was administered i.p. daily at 20 µg/dose per mouse, starting one day before laser induction to day 3 post laser injury (d-1, d0, d1, d2 and d3).

### Flow cytometry

Enucleated eyes were prepared under a dissection microscope, and retina and RPE/choroid were processed separately. Retinal infiltrating cells were isolated by physical disruption of dissected retinas before passing cell suspensions through a 40-µm cell strainer using a syringe plunger, washed and resuspended in staining buffer (balanced salt solution with 0.1% BSA and 0.08% sodium azide). For isolation of RPE/choroidal cells, tissue was digested in Hank’s Balance Salt Solution (PAA) containing 0.5 mg/ml collagenase D, and 750 U/ml DNase I (Sigma-Aldrich). After 20 minutes at 37^o^C, an additional 0.5 mg/ml collagenase D and 750 U/ml DNase I were added. The mixture was then incubated for an additional 10 minutes at 37^o^C, before being passed through a cell strainer and resuspended as for retina. Single-cell suspensions from pooled retinal or RPE/choroidal samples from three eyes were incubated with anti-CD16/32 (2.4G2) mAb for 10 minutes at 4^o^C before incubation with fluorochrome-conjugated mAbs against cell surface markers including CD45 and F4/80 or isotype antibodies at 4^o^C for 20 minutes. To determine MC-21 depletion efficacy, whole blood collected from the tail vein was incubated, with MC-21 or isotype control (Rat IgG2b) for 1 hour on ice. Cells were then washed three times with ice-cold PBS, before second incubation for 30 minutes on ice with biotinylated mouse anti-rat IgG2b mAb. Cells were then blocked with 10% rat serum, before incubation with streptavidin-APC, and directly conjugated mAbs against Ly6C, F4/80, CD11b and CD45 surface markers. Cell suspensions were acquired with a LSR-II flow cytometer (BD Cytometry Systems) and analysis performed using FlowJo software version 7.0 (Treestar).

### CD11b^+^ cell enrichment by magnetic-activated cell sorting (MACS)

Microglia and myeloid cell populations were isolated by enriching CD11b^+^ cells from single-cell suspensions of RPE/choroid or retina tissues pooled from four eyes, using anti-CD11b microbeads (Miltenyi Biotec) according to the manufacturer’s instruction. Both CD11b^-^ and CD11b^+^ cell fractions were collected and prepared for RNA isolation and QRT-PCR analysis.

### Generation of necrotic or apoptotic cells

Necrotic RPE cells (RPEs) were generated from a mouse RPE cell line B6-RPE07 [66] or a human RPE cell line ARPE19 (American Type Culture Collection) by heating a known number of cells for 15 minutes at 95°C, and cell death was confirmed by trypan blue exclusion. Apoptotic RPEs were induced by incubation in serum-free medium containing different concentrations of hydrogen peroxide (H_2_O_2_, 0.5-2 mM) for 24 hours at 37 ^o^C [67]. Cell apoptosis was confirmed by microscopic observation of nuclear condensation (DAPI staining) and flow cytometric analysis using annexin V-APC/7AAD staining (BD Biosciences).

For isolation of mouse retinal vascular endothelial cells, 1 ml of pooled retinal single-cell suspension from 14 retinas was incubated with rat anti-CD31 (2 µg, BD Biosciences) in PBS with 0.5% BSA at RT for 15 minutes. After wash, samples were then incubated with Dynabeads Sheep anti-rat IgG (Life Technologies) for 20 minutes, followed by separation of the vessel-enriched fraction using a magnetic separator according to manufacturer’s instruction (Life Technologies). Neutrophils were isolated from mouse bone marrow based on negative-selection using a Neutrophil Isolation Kit (Miltenyi Biotec). Necrotic cells prepared from the retinal endothelial cells, neutrophils, B cells (YTS156 hybridoma, a gift from Dr Awen Gallimore, Cardiff University) and whole retinal cells were generated as above.

### Mouse bone marrow-derived macrophages, human microglia cell line and treatment

Mouse primary bone marrow-derived macrophages (BMMΦs) were generated from C57BL/6J mice as reported in our previous studies [29]. A human microglia cell line C13-NJ was a kind gift from Prof. A. Randall (University of Bristol) and was cultured and maintained as described previously [68]. Cells were plated at a concentration of 1×10^6^ cells/well in 24-well plates and incubated for 3 hours to allow cell attachment. Cell culture supernatant was replaced with serum-free DMEM medium containing either necrotic or apoptotic cells, for an additional 24 hours incubation. After wash, total RNA from mouse BMMΦs or human microglia treated with necrotic cells was harvested in TRIzol for QRT-PCR analysis. As for analysis of BMMΦs treated with apoptotic RPEs, to avoid contamination from any remaining live RPE cells in co-culture of BMMΦs and apoptotic cells, CD11b^+^ cells were MACS isolated and purity of the macrophages was confirmed by F4/80-PE staining and flow cytometry before subsequent gene expression analysis.

### In vitro phagocytosis

B6-RPE07 cells were labelled with Vybrant CFDA SE Cell Tracer (Life Technologies) according to manufacturer’s instruction, before generation of necrotic or apoptotic cell suspensions, which were then added to CellTrace Violet-labelled BMMΦs grown on cell culture slides. After 1 hour incubation, cells were washed in PBS to remove un-internalised RPEs and debris, followed by fixation with 4% PFA. Slides were mounted using antifade medium for confocal microscopy.

### VEGF ELISA

To measure VEGF secretion from mouse BMMΦs treated with necrotic RPEs, we removed un-internalised necrotic RPE/debris and supernatant after 60 minutes of incubation by washing 3 times with fresh culture medium to deprive any VEGF released from dead RPEs. After a further 24 hour-incubation in serum-free medium, supernatant from the macrophage culture was harvested and stored at -80°C until analysis for VEGF concentrations by a sandwich ELISA kit (R&D Systems).

### Statistics

Results are presented as means ± SEM. Data were analysed using Student’s two-tailed *t* test as described previously [18,19]. Differences between groups were considered significant at *P*<0.05.

## Supporting Information

Figure S1 **Development of angiogenesis and CNV formation.** Six to eight-week-old male mice were induced for CNV by laser photocoagulation. Eyes were taken at indicated time points between 3 hours and 14 days post laser induction. RPE/choroidal tissues were separated for isolectin B4 (IB4) staining and whole-mounted for confocal microscopy. Representative images show laser-induced development of choroidal angiogenesis.
(TIFF)

Figure S2 **Time course of inflammation-associated gene expression is mainly produced by accumulating macrophages.** (**A**) QRT-PCR analysis of time-dependent *Nos2*, *Tnfα*, *Il1β* and *Cd200r* expression in RPE/choroid and retina tissues. (**B**) Cellular gene expression on day 2 using CD11b MACS-isolated cells pooled from 4 eyes showing inflammatory *Il1β* and *Tnfα* gene expression largely produced by myeloid cells. Data are presented as mean ± SEM, n=3-6 per time point. ^*^ *P*<0.05 vs. control. ND, not detected.
(TIFF)

Figure S3 **Confirmation of Arginase-1 expression by accumulating macrophages in RPE/choroid.** (**A**) RPE/choroid tissues were collected 2 days post laser and single-cell suspensions prepared. CD11b^+^ and CD11b^-^ cells were isolated via MACS and equal amount of cellular RNA from each sample was analysed by conventional RT-PCR, showing definitive *Arg-1* expression in CD11b^+^ cell population from laser-treated RPE/choroid. It is noted that, although either CD11b^+^ or CD11b^-^ cells show similar housekeeping gene *Gapdh* levels between normal and laser-treated samples, CD11b^-^ cells have greater *Gapdh* mRNA level compared with CD11b^+^ cells. Negative control using nuclease-free sterile water yielded no amplification product. (**B**) Confocal images demonstrate presence of CD11b and Arg-1 double-positive cells at lesions on RPE/choroidal whole-mounts collected on day 3 post laser.
(TIFF)

Figure S4 **Early but not exclusive VEGF expression by lesional macrophages.** RPE/choroid tissues were collected at different time points post laser induction, stained for Iba1 and VEGF, and observed using confocal microscopy. (**A**) Resident retinal macrophages (microglia) at the RPE/choroid interface have no significant VEGF immuno-reactivity. (**B**) On day 1, both ramified microglia and amoeboid macrophages at the site of laser injury display VEGF positivity (**yellow arrow**). By day 2 (C) and 4 (D), lesional macrophages express greater VEGF (**yellow arrow**) and VEGF-expressing Iba1-negative cells are also observed within lesion on day 4. VEGF immuno-reactivity within macrophages diminishes after 7 days (**E**, **red arrow**) at the time the substantial angiogenic buds are established, and no macrophage VEGF expression is evident by day 14 (**F**, **red arrow**).
(TIFF)

Figure S5 **Plot profiles of Iba1 and VEGF immuno-reactivity on RPE/choroid lesions day 1 post laser induction.** RPE/choroid tissues were collected on day 1 post laser and stained for Iba1 and VEGF. Representative confocal images and analysis of plot profiles using ImageJ (version 1.28u) demonstrate similar distribution of intensity peaks of pixels between Iba1 and VEGF immune-fluorescence (**red double-arrow**) along a rectangular selection at the lesion area.
(TIFF)

Figure S6 **Systemic depletion of CCR2^+^ monocytes results in loss of CCR2^+^ cells at the site of lesion on day 2 post laser induction.** Anti-CCR2 mAb (MC-21) or isotype antibody was administered (i.p.) at 20 µg per mouse daily from one day before laser induction. RPE/choroidal tissues were collected on day 2 post laser and immuno-stained with a rat monoclonal anti-CD11b-biotin and goat polyclonal anti-CCR2, followed by detection with Rhodamine Red-X-labelled streptavidin and Alexa Fluor 488-conjugated rabbit anti-goat IgG, respectively. Representative confocal images show the loss of specific CCR2 immuno-reactivity in accumulating CD11b^+^ cells at site of injury in MC-21 treated animals, compared with isotype antibody administrated controls.
(TIFF)

Figure S7 **Accumulating macrophages are endocytic, engulfing fragments of damaged RPE.** (**A**) RPE/choroid were stained for Iba1 and analysed by confocal microscopy. Representative confocal images show ramified microglia within normal tissue, and amoeboid activated macrophages at lesion site demonstrated by surface ruffling (**arrow**), a sign of cell phagocytic activity. (**B**) Bright field and fluorescence confocal images show pigment-engulfing macrophages at lesion site from the retina side, where bright field images were colour-processed from black(pigment)/white(retina) to green(pigment)/black(retina) and then merged with Iba1 staining (red). (**C**) *Ex vivo* macrophage engulfment in laser lesion on retina side. Post-laser retinas were isolated and cultured with CD11b mAb (green) and pHrodo Red-Dextran which become fluorescent once in endosome. Internalisation and processing of the conjugate reagent in CD11b^+^ cells close to the lesion were seen after 40 minutes (**arrow**). After 3-24 hours, more significant fluorescent pHrodo Red was detected within the accumulating macrophages (**arrow**). Blue, hoechst stain. Bar, 20 µm (**A**) or 10 µm (**B** and **C**).
(TIFF)

Figure S8 **Apoptotic RPE mediates macrophage phenotype.** (**A**) Apoptotic B6-RPE07 cells were generated with oxidative stress by incubation with 1 mM of H_2_O_2_ for 24 hours. Annexin V/7AAD dual staining of RPE cells and flow cytometry were used to analyse populations undergoing early or late apoptosis. (**B**) Following 60 minutes of co-culture with apoptotic RPE cells (CFDA-labelled), BMMΦs (Violet Tracer-labelled) engulf damaged RPE cells/debris, as evident by top and side views of confocal images. After 24 hours of incubation with apoptotic RPE cells, BMMΦs were isolated using CD11b-MACS and analysed by QRT-PCR for gene expression of *Arg-1* and *Nos2* (**C**), and *Vegf* (**D**). Data are presented as mean ± SEM, n=3. *18s rRNA* was used as an internal control. Ratio stands for number of RPE cells to macrophages.
(TIFF)

Figure S9 **MAC deposition exaggerates at the time myeloid cells accumulate at site of injury.** To examine the kinetics of local complement activation in response to laser trauma, RPE/choroid was collected at different time points post laser and immunostained with the antibody against C9 (a marker for membrane attack complex, MAC). Representative confocal images demonstrate that MAC deposits rapidly at the laser site within 3 hours, strongly exaggerates after day 1, remained at high levels until day 4. Thereafter MAC deposition decreases during days 7-14.
(TIFF)
