# Supplementary figures and images for: Myeloid Cells Expressing VEGF and Arginase-1 Following Uptake of Damaged Retinal Pigment Epithelium Suggests Potential Mechanism That Drives the Onset of Choroidal Angiogenesis in Mice
Source: PLoS One. 2013 Aug 16;8(8):e72935. doi: 10.1371/journal.pone.0072935 (PMC3745388; doi:10.1371/journal.pone.0072935)

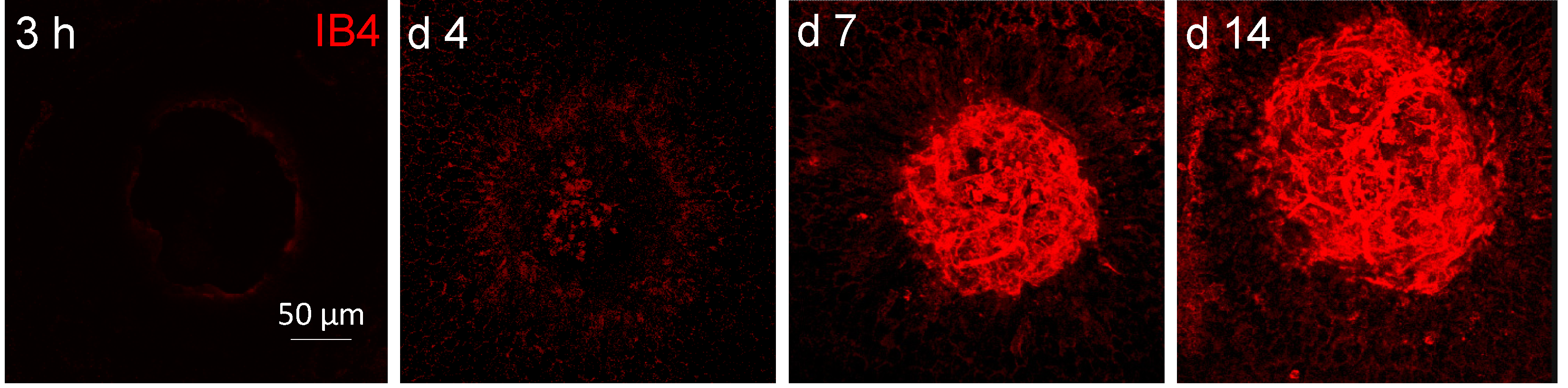

Supplement: Figure S1 — Development of angiogenesis and CNV formation. Six to eight-week-old male mice were induced for CNV by laser photocoagulation. Eyes were taken at indicated time points between 3 hours and 14 days post laser induction. RPE/choroidal tissues were separated for isolectin B4 (IB4) staining and whole-mounted for confocal microscopy. Representative images show laser-induced development of choroidal angiogenesis. (TIFF) [file pone.0072935.s001.tiff]

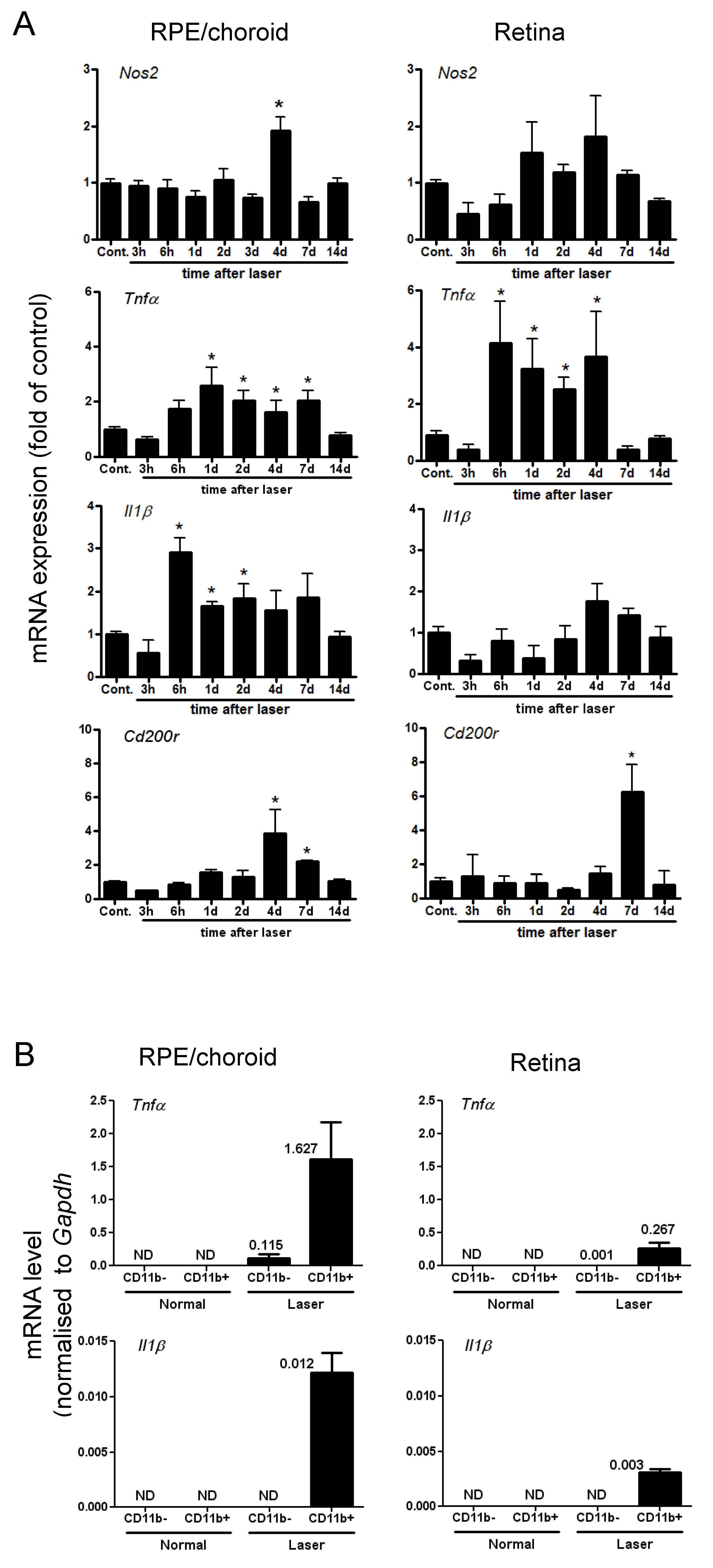

Supplement: Figure S2 — Time course of inflammation-associated gene expression is mainly produced by accumulating macrophages. (A) QRT-PCR analysis of time-dependent Nos2, Tnfα, Il1β and Cd200r expression in RPE/choroid and retina tissues. (B) Cellular gene expression on day 2 using CD11b MACS-isolated cells pooled from 4 eyes showing inflammatory Il1β and Tnfα gene expression largely produced by myeloid cells. Data are presented as mean ± SEM, n=3-6 per time point. * P<0.05 vs. control. ND, not detected. (TIFF) [file pone.0072935.s002.tiff]

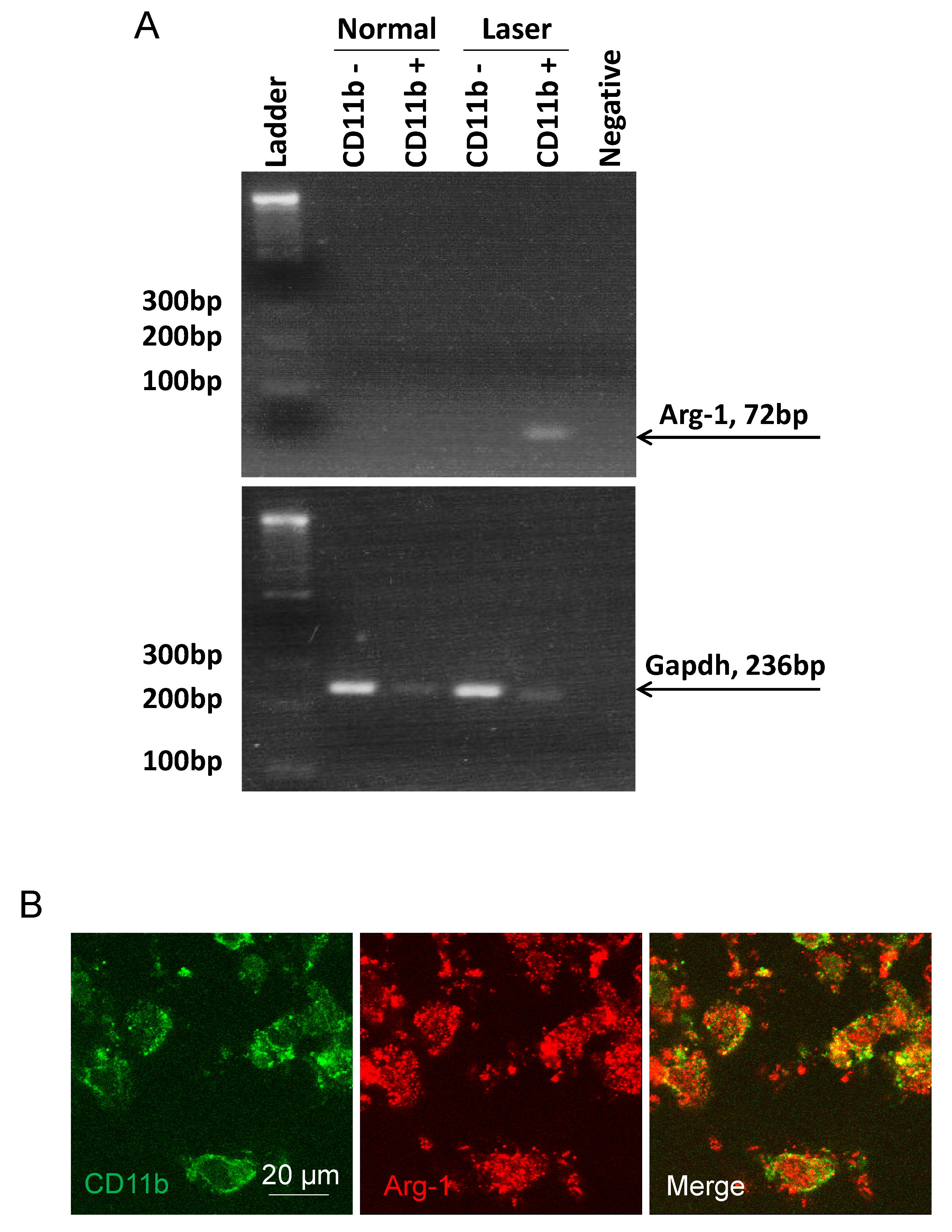

Supplement: Figure S3 — Confirmation of Arginase-1 expression by accumulating macrophages in RPE/choroid. (A) RPE/choroid tissues were collected 2 days post laser and single-cell suspensions prepared. CD11b+ and CD11b- cells were isolated via MACS and equal amount of cellular RNA from each sample was analysed by conventional RT-PCR, showing definitive Arg-1 expression in CD11b+ cell population from laser-treated RPE/choroid. It is noted that, although either CD11b+ or CD11b- cells show similar housekeeping gene Gapdh levels between normal and laser-treated samples, CD11b- cells have greater Gapdh mRNA level compared with CD11b+ cells. Negative control using nuclease-free sterile water yielded no amplification product. (B) Confocal images demonstrate presence of CD11b and Arg-1 double-positive cells at lesions on RPE/choroidal whole-mounts collected on day 3 post laser. (TIFF) [file pone.0072935.s003.tiff]

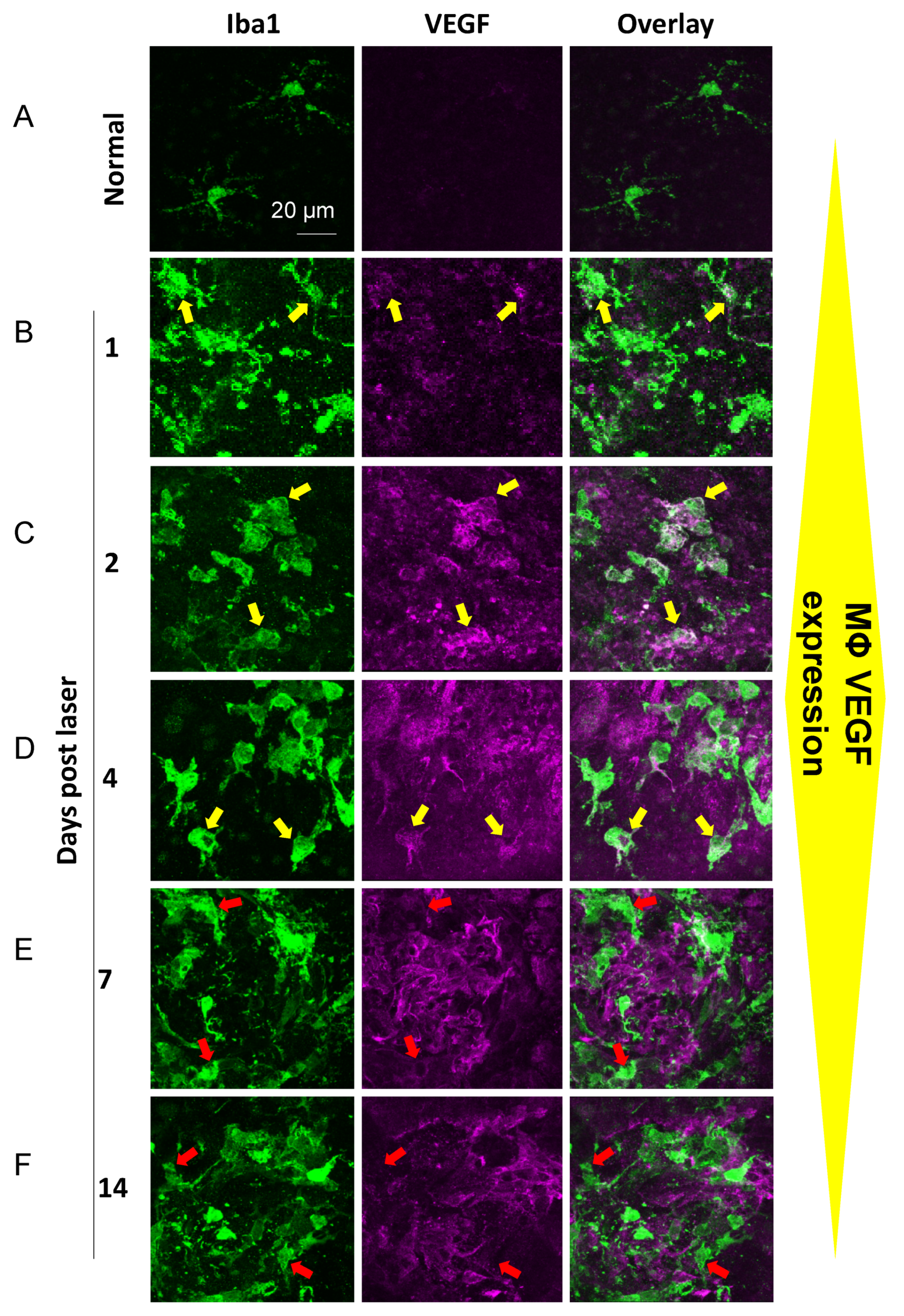

Supplement: Figure S4 — Early but not exclusive VEGF expression by lesional macrophages. RPE/choroid tissues were collected at different time points post laser induction, stained for Iba1 and VEGF, and observed using confocal microscopy. (A) Resident retinal macrophages (microglia) at the RPE/choroid interface have no significant VEGF immuno-reactivity. (B) On day 1, both ramified microglia and amoeboid macrophages at the site of laser injury display VEGF positivity (yellow arrow). By day 2 (C) and 4 (D), lesional macrophages express greater VEGF (yellow arrow) and VEGF-expressing Iba1-negative cells are also observed within lesion on day 4. VEGF immuno-reactivity within macrophages diminishes after 7 days (E, red arrow) at the time the substantial angiogenic buds are established, and no macrophage VEGF expression is evident by day 14 (F, red arrow). (TIFF) [file pone.0072935.s004.tiff]

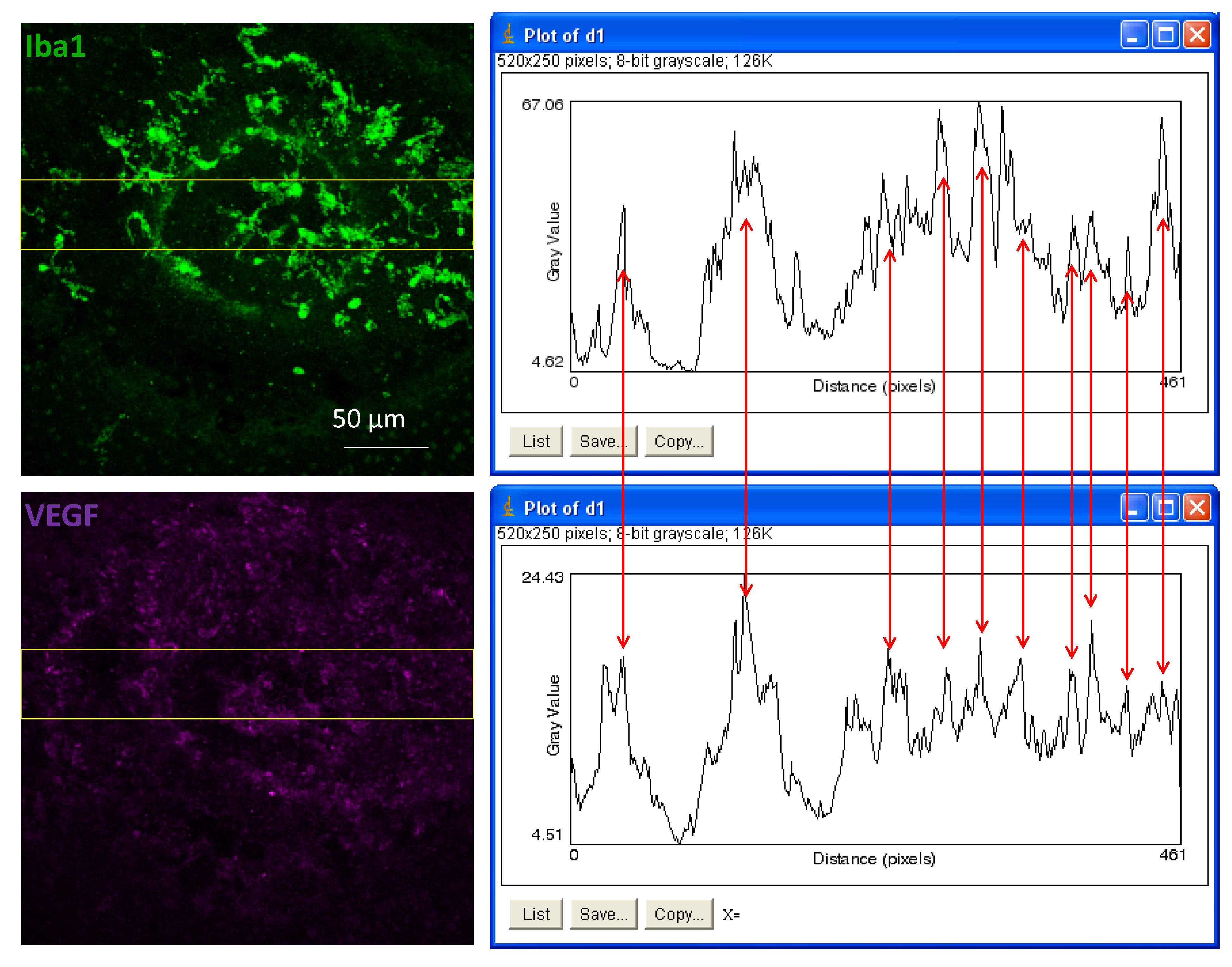

Supplement: Figure S5 — Plot profiles of Iba1 and VEGF immuno-reactivity on RPE/choroid lesions day 1 post laser induction. RPE/choroid tissues were collected on day 1 post laser and stained for Iba1 and VEGF. Representative confocal images and analysis of plot profiles using ImageJ (version 1.28u) demonstrate similar distribution of intensity peaks of pixels between Iba1 and VEGF immune-fluorescence (red double-arrow) along a rectangular selection at the lesion area. (TIFF) [file pone.0072935.s005.tiff]

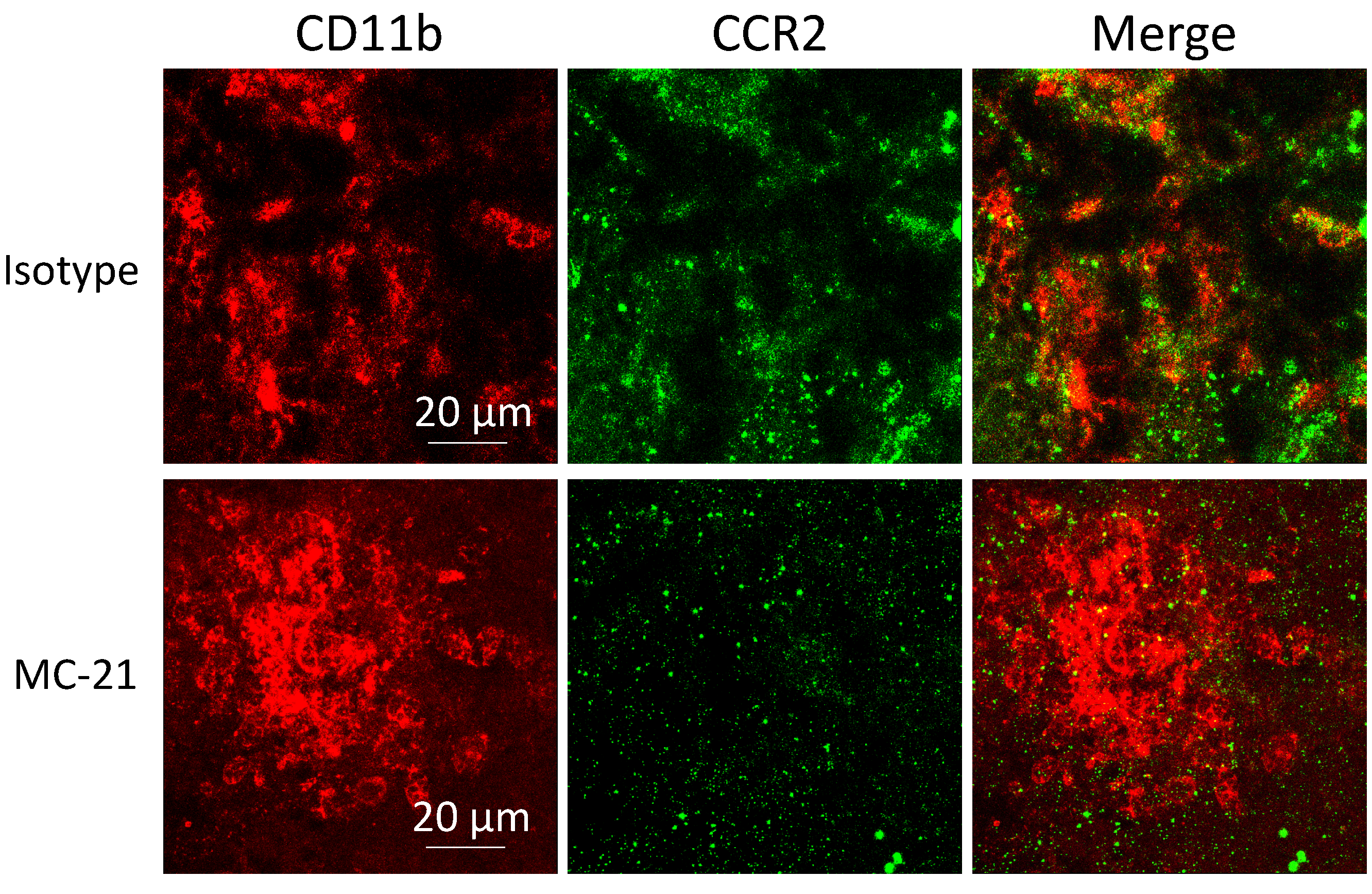

Supplement: Figure S6 — Systemic depletion of CCR2+ monocytes results in loss of CCR2+ cells at the site of lesion on day 2 post laser induction. Anti-CCR2 mAb (MC-21) or isotype antibody was administered (i.p.) at 20 µg per mouse daily from one day before laser induction. RPE/choroidal tissues were collected on day 2 post laser and immuno-stained with a rat monoclonal anti-CD11b-biotin and goat polyclonal anti-CCR2, followed by detection with Rhodamine Red-X-labelled streptavidin and Alexa Fluor 488-conjugated rabbit anti-goat IgG, respectively. Representative confocal images show the loss of specific CCR2 immuno-reactivity in accumulating CD11b+ cells at site of injury in MC-21 treated animals, compared with isotype antibody administrated controls. (TIFF) [file pone.0072935.s006.tiff]

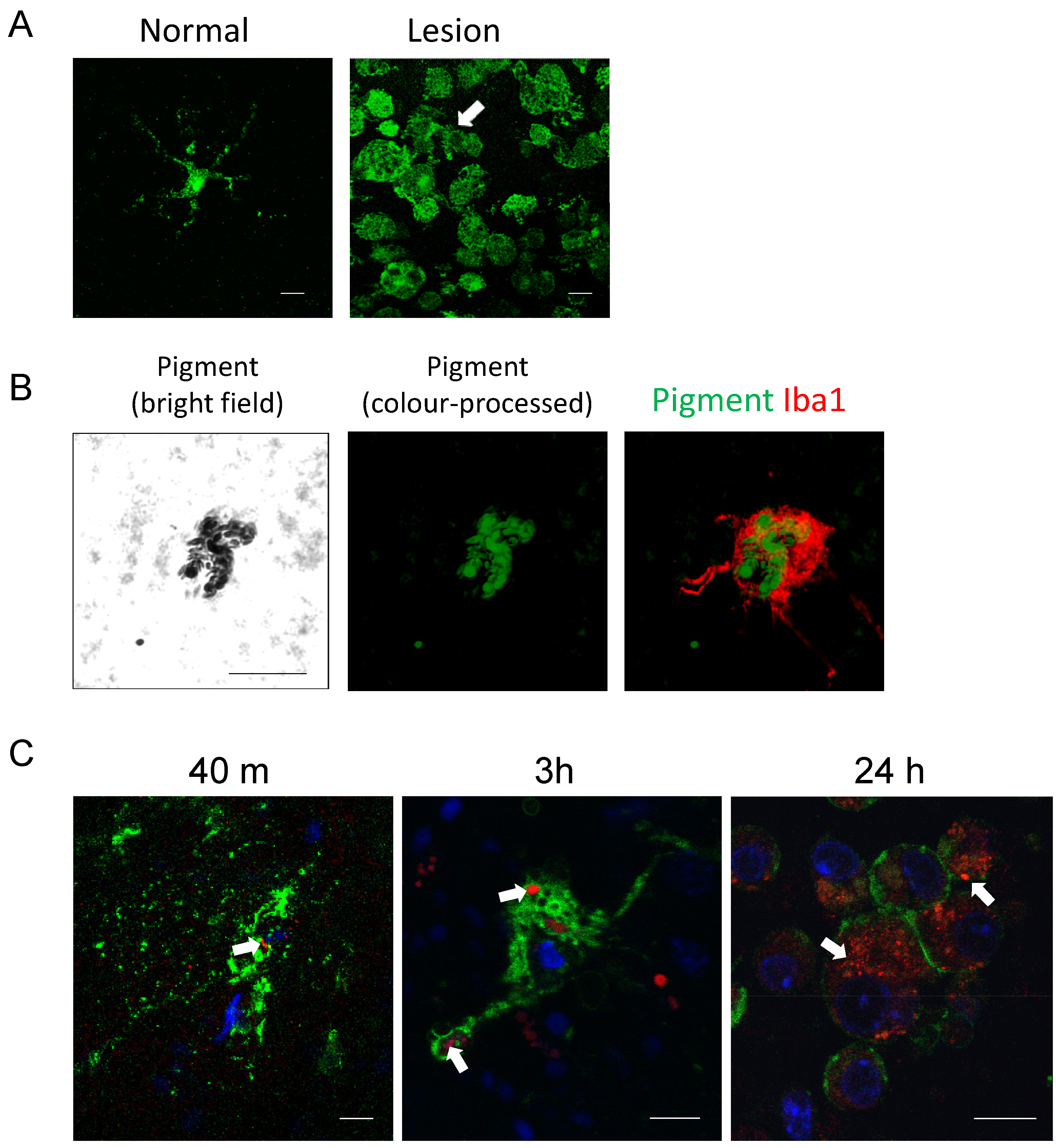

Supplement: Figure S7 — Accumulating macrophages are endocytic, engulfing fragments of damaged RPE. (A) RPE/choroid were stained for Iba1 and analysed by confocal microscopy. Representative confocal images show ramified microglia within normal tissue, and amoeboid activated macrophages at lesion site demonstrated by surface ruffling (arrow), a sign of cell phagocytic activity. (B) Bright field and fluorescence confocal images show pigment-engulfing macrophages at lesion site from the retina side, where bright field images were colour-processed from black(pigment)/white(retina) to green(pigment)/black(retina) and then merged with Iba1 staining (red). (C) Ex vivo macrophage engulfment in laser lesion on retina side. Post-laser retinas were isolated and cultured with CD11b mAb (green) and pHrodo Red-Dextran which become fluorescent once in endosome. Internalisation and processing of the conjugate reagent in CD11b+ cells close to the lesion were seen after 40 minutes (arrow). After 3-24 hours, more significant fluorescent pHrodo Red was detected within the accumulating macrophages (arrow). Blue, hoechst stain. Bar, 20 µm (A) or 10 µm (B and C). (TIFF) [file pone.0072935.s007.tiff]

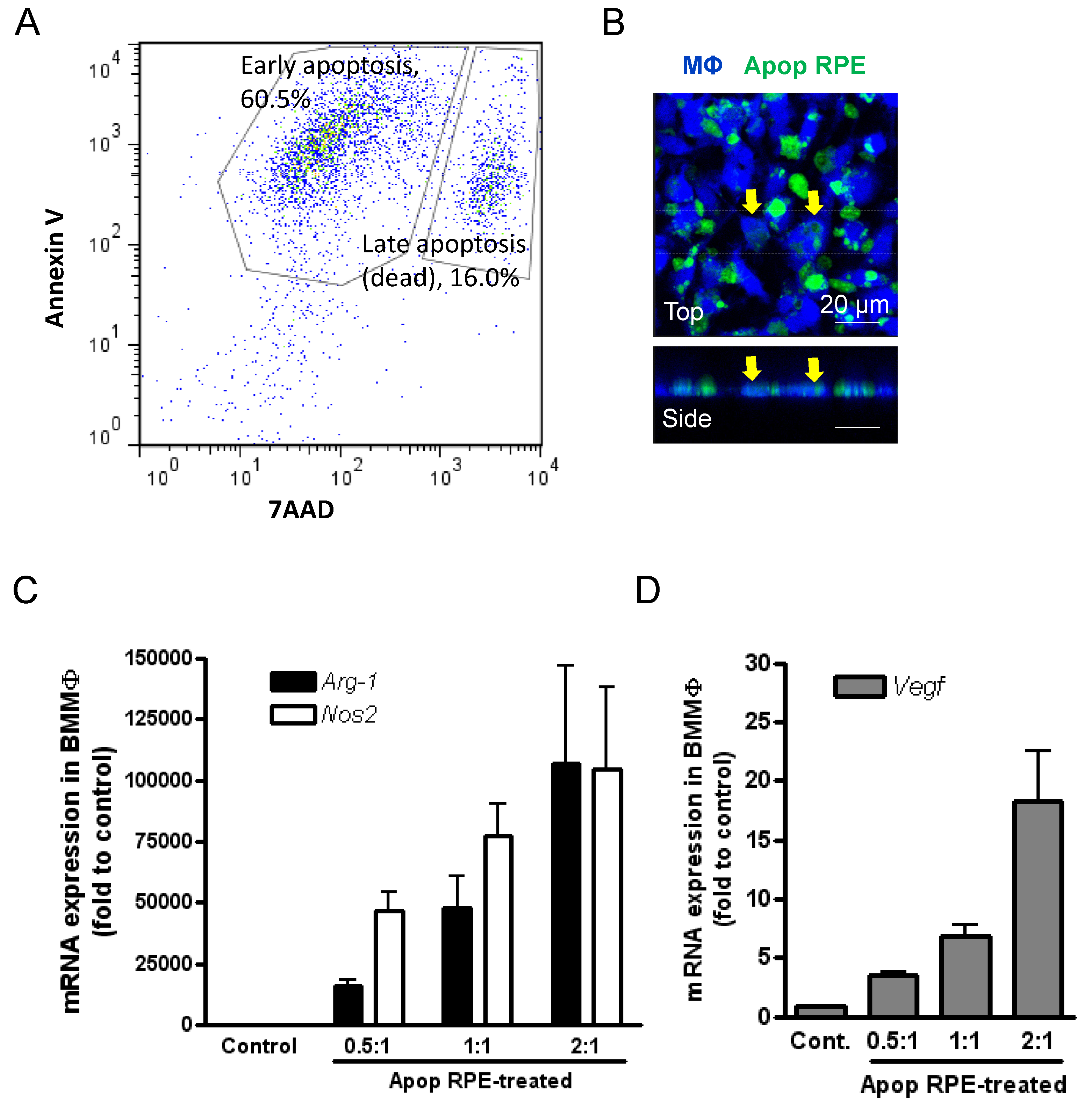

Supplement: Figure S8 — Apoptotic RPE mediates macrophage phenotype. (A) Apoptotic B6-RPE07 cells were generated with oxidative stress by incubation with 1 mM of H2O2 for 24 hours. Annexin V/7AAD dual staining of RPE cells and flow cytometry were used to analyse populations undergoing early or late apoptosis. (B) Following 60 minutes of co-culture with apoptotic RPE cells (CFDA-labelled), BMMΦs (Violet Tracer-labelled) engulf damaged RPE cells/debris, as evident by top and side views of confocal images. After 24 hours of incubation with apoptotic RPE cells, BMMΦs were isolated using CD11b-MACS and analysed by QRT-PCR for gene expression of Arg-1 and Nos2 (C), and Vegf (D). Data are presented as mean ± SEM, n=3. 18s rRNA was used as an internal control. Ratio stands for number of RPE cells to macrophages. (TIFF) [file pone.0072935.s008.tiff]

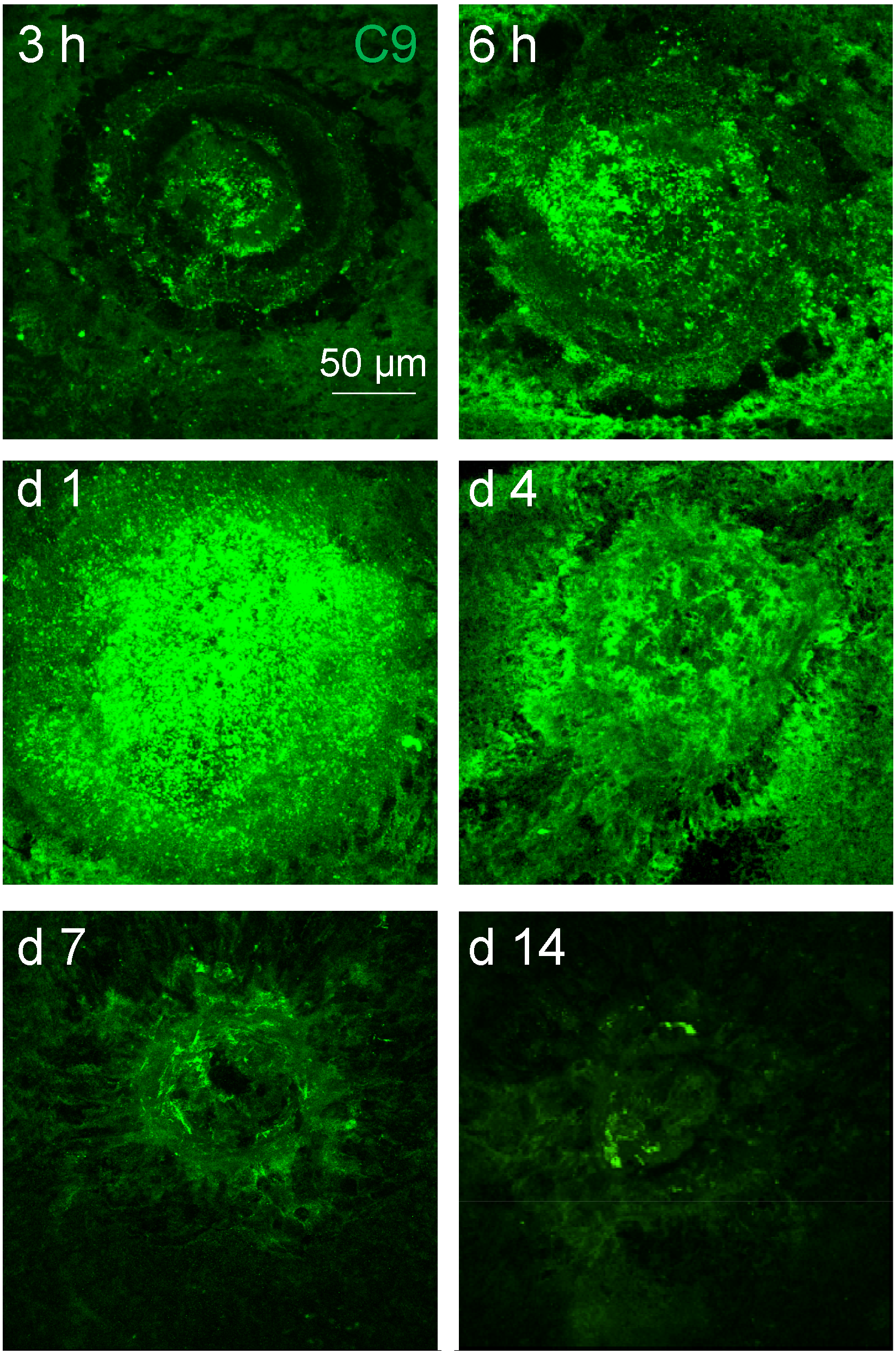

Supplement: Figure S9 — MAC deposition exaggerates at the time myeloid cells accumulate at site of injury. To examine the kinetics of local complement activation in response to laser trauma, RPE/choroid was collected at different time points post laser and immunostained with the antibody against C9 (a marker for membrane attack complex, MAC). Representative confocal images demonstrate that MAC deposits rapidly at the laser site within 3 hours, strongly exaggerates after day 1, remained at high levels until day 4. Thereafter MAC deposition decreases during days 7-14. (TIFF) [file pone.0072935.s009.tiff]
